# Prenatal and early-life environmental factors, family demographics and cortical brain anatomy in 5-year-olds: an MRI study from FinnBrain Birth Cohort

**DOI:** 10.1007/s11682-022-00679-w

**Published:** 2022-07-22

**Authors:** Eero Silver, Elmo P. Pulli, Eeva-Leena Kataja, Venla Kumpulainen, Anni Copeland, Ekaterina Saukko, Jani Saunavaara, Harri Merisaari, Tuire Lähdesmäki, Riitta Parkkola, Linnea Karlsson, Hasse Karlsson, Jetro J. Tuulari

**Affiliations:** 1 FinnBrain Birth Cohort Study, Turku Brain and Mind Center, Department of Clinical Medicine, University of Turku, Lemminkäisenkatu 3, FinnBrain Study, Teutori Building, 2nd floor, 21520 Turku, Finland; 2 Department of Psychology, University of Turku, Turku, Finland; 3 Department of Radiology, Turku University Hospital, Turku, Finland; 4 Department of Medical Physics, University of Turku and Turku University Hospital, Turku, Finland; 5 Department of Pediatric Neurology, Turku University Hospital, University of Turku, Turku, Finland; 6 Department of Radiology, Turku University Hospital, University of Turku, Turku, Finland; 7 Centre for Population Health Research, Turku University Hospital and University of Turku, Turku, Finland; 8 Department of Psychiatry, Turku University Hospital & University of Turku, Turku, Finland; 9 Department of Peadiatrics and Adolescent Medicine, Turku University Hospital & University of Turku, Turku, Finland; 10 Turku Collegium for Science, Medicine and Technology, University of Turku, Turku, Finland

**Keywords:** MRI, Brain, Cortical development, Child

## Abstract

The human brain develops dynamically during early childhood, when the child is sensitive to both genetic programming and extrinsic exposures. Recent studies have found links between prenatal and early life environmental factors, family demographics and the cortical brain morphology in newborns measured by surface area, volume and thickness. Here in this magnetic resonance imaging study, we evaluated whether a similar set of variables associates with cortical surface area and volumes measured in a sample of 170 healthy 5-year-olds from the FinnBrain Birth Cohort Study. We found that child sex, maternal pre-pregnancy body mass index, 5 min Apgar score, neonatal intensive care unit admission and maternal smoking during pregnancy associated with surface areas. Furthermore, child sex, maternal age and maternal level of education associated with brain volumes. Expectedly, many variables deemed important for neonatal brain anatomy (such as birth weight and gestational age at birth) in earlier studies did not associate with brain metrics in our study group of 5-year-olds, which implies that their effects on brain anatomy are age-specific. Future research may benefit from including pre- and perinatal covariates in the analyses when such data are available. Finally, we provide evidence for right lateralization for surface area and volumes, except for the temporal lobes which were left lateralized. These subtle differences between hemispheres are variable across individuals and may be interesting brain metrics in future studies.

**Supplementary Information:**

The online version contains supplementary material available at 10.1007/s11682-022-00679-w.

## Introduction

The cortical brain development is fast during the first years of life (Knickmeyer et al., 2008), and this trend is reflected in all typically used metrics such as cortical thickness (CT), surface area (SA), gyrification and cortical volumes (Remer et al., 2017). These metrics seem to capture distinct patterns of brain maturation, and indeed, are frequently discussed separately in recent studies (Kuhl et al., 2020; Lyall et al., 2015; Yang et al., 2016). Along with rapid structural growth of the brain during the first years of life, hemispheric asymmetry with non-linear developmental trajectories is a phenomenon commonly reported in magnetic resonance imaging (MRI) studies (Ball et al., 2012; Toga & Thompson, 2003).

Cortical development is sensitive to extrinsic factors starting from the perinatal period, and these exposures may have long-lasting and often adverse effects on the neurocognitive development among the vulnerable individuals later in life (Duan et al., 2019; Lehtola et al., 2019; Pulli et al., 2019). In a broader context, many psychiatric and neurocognitive disorders have been postulated to result from alterations in cortical and subcortical maturation in the early childhood (Hazlett et al., 2012; Li et al., 2016), and to capture such signals is of importance for both clinical and scientific purposes. For example, the ability to select an appropriate set of covariates for the data analyses, is dependent on the knowledge of important and potentially confounding factors.

A study by Jha et al. (2019) observed the effects of obstetric and prenatal environmental factors on CT and SA in newborns. Average CT correlated positively with postnatal age at MRI and negatively with paternal education. In addition, maternal ethnicity associated with the average CT, with the offspring of African American mothers presenting larger CT than Caucasian mothers. Total SA showed positive correlations with birth weight, gestational age at birth and postnatal age at MRI. Furthermore, males were found to have significantly greater total SA than females. These results demonstrate the role of perinatal surroundings and family demographics for early cortical anatomy (Sheridan et al., 2012; Wu et al., 2020). These results also suggest that the early organization of cortical structure is affected by both maternal and paternal factors, that may be genetic. To which extent these environmentally associated structural features persist to later ages during childhood is still unclear.

In the current study, we quantified the associations between demographics and gross anatomy of cortical SA and volumes in healthy, typically developing 5-year-olds, and discussed how these results were in line with prior work (Jha et al., 2019). The study by Jha et al. (2019) was selected as the prior reference work due to its unique and large newborn dataset, which is a combination of comprehensive background information and quality MRI data of the participants. If the demographics that have been shown to be associated with neonatal brain anatomy still explained later childhood brain features, it would imply the presence of possible programming effect. It would also suggest that the perinatal time period has a crucial role in cortical development considering the following years of life, too. However, we generally expected to find that most of these factors that associated with newborn brain anatomy (e.g. birth weight, gestational age at birth and postnatal age at MRI) in the previous study do not explain structural features in 5-year-olds, which could mean that postnatal environment and genetics are prominent determinants of early childhood brain development. On the other hand, we expected to find sex differences and demographics that are continuous exposures from the viewpoint of the child, such as maternal socioeconomic status (SES) would be associated with brain morphology through the first five years of life. In addition, we provided descriptive statistics on cortical lateralization.

## Methods

The study was conducted according to the Declaration of Helsinki and was reviewed and approved by the Ethics Committee of the Hospital District of Southwest Finland (ETMK:31/180/2011).

### Participants

The participants of the current MRI study are part of a larger FinnBrain Birth Cohort Study population (www.finnbrain.fi) (Karlsson et al., 2018). Initially, mothers and their spouses were recruited at three maternal welfare clinics in Southwest Finland during the first trimester ultrasound visits. The participating mothers of the current sample were mostly of Finnish background. Parental background information was gathered during pregnancy by questionnaires and included monthly income, educational level, diagnosed medical conditions, medications affecting central nervous system (CNS), and substance use during pregnancy. For CNS affecting medications, only serotonin reuptake inhibitors and norepinephrine reuptake inhibitors (SSRI/SNRI) or benzodiazepines were reported. No illicit drug use substance was reported in the current sample. Parental psychiatric history variables were determined based on questionnaires for mothers, that screened the parents for symptoms of depression, anxiety disorder and psychotic symptoms. Obstetric data (neonatal intensive care unit (NICU) admission, 5 min Apgar score, Maternal BMI before gestation, Diagnosis of gestation diabetes) were retrieved from the Finnish Medical Birth Register of the National Institute for Health and Welfare (www.thl.fi).

The inclusion criteria for the imaging measurements were a visit to *FinnBrain Child Development and Parental Functioning Lab*, including neuropsychological measurements, at 5 years of age. However, five participants were included without neuropsychological visits: three participants had an exposure to maternal prenatal synthetic glucocorticoid treatment (recruited separately for a nested case–control sub-study). In addition, two participants were enrolled for pilot scans. The exclusion criteria specific for this study were: 1) born before gestational week 35, 2) developmental anomaly or abnormalities in senses or communication (e.g. blindness, deafness, congenital heart disease), 3) known long-term medical diagnosis (e.g. epilepsy, autism), 4) ongoing medical examinations or clinical follow up in a hospital (meaning there has been a referral from primary care setting to special health care), 5) child use of continuous, daily medication (including per oral medications, topical creams and inhalants. Exceptions to this were desmopressin (®Minirin) medication and asthma inhalers during infection, which were allowed), 6) history of head trauma reported by parents (defined as concussion necessitating clinical follow up in a health care setting or worse), 7) metallic (golden) ear tubes, and routine MRI contraindications.

The MRI scans were performed between 29 October 2017 and 1 March 2021. Altogether 541 families were contacted and 478 (88%) of them reached. In total, 203 (42% of the reached families) participants attended imaging visits.

### MRI data acquisition

All MRI scans were performed for research purposes by the research staff. Before the visit, each family was contacted and recruited via telephone calls by a research staff member. A written informed consent was acquired from both parents at the beginning of the MRI visit. Before the imaging, a research member met families personally to share information about the study visit and to bring practice materials for home rehearsal. During the study visit, a flexible timetable was reserved for the subjects to get familiar with the research staff. Imaging was practiced with a mock scanner (incl. wooden head coil) and a light meal was served before the scan.

The subjects were scanned during natural sleep or awake while they were watching a movie or TV show of their choice. Hearing protection included wax earplugs and headphones. Foam padding was applied to help the head stay still and assure comfortable position. A member of the research staff and a parent stayed in the scanner room throughout the scan. Subjects were given a “signal ball” to throw in case they needed or wanted to stop the scan at any point. The protocol of the preparations and the study visit are described in detail in our previous studies (Copeland et al., 2021; Pulli et al., 2021).

Subjects were scanned with a Siemens Magnetom Skyra fit 3 T (Siemens Medical Solutions, Erlangen, Germany) using a 20-element head/neck coil. The Generalized Autocalibrating Partially Parallel Acquisition (GRAPPA) technique was used to accelerate image acquisition (Parallel acquisition techniques factor of 2 was used). The MRI data was acquired as a part of max. 60-min scan protocol. For the purposes of the current study, we acquired high resolution 3D T1 images using magnetization prepared rapid gradient echo (MPRAGE) sequence with following sequence parameters: TR = 1900 ms, TE = 3.26 ms, TI = 900 ms, flip angle = 9 degrees, voxel size = 1.0 × 1.0 × 1.0 mm3, FOV 256 × 256 mm2. In addition, MRI protocol consisted of a T2 turbo spin echo (TSE), a 96-direction (b = 1000 s/mm2, 9 b0s, TR/TE = 9300/87.0 ms) diffusion tensor imaging (DTI) sequences (Merisaari et al., 2019) and a 7-min functional MRI acquisition.

### Image analysis

Three out of the 203 participants that attended the imaging visit were born before 35 weeks of gestation and 13 subjects were excluded due to the failure or excess motion artefact in the T1-weighted images. Thereafter, 187 subjects’ T1-weighted images entered the processing pipeline (Pulli et al., 2021) and after assessing the automated segmentation, 170 were qualified into the analyses. Cortical reconstruction and volumetric segmentation for all images that were included to the processing pipeline was performed with the FreeSurfer software suite, version 6.0.0 (http://surfer.nmr.mgh.harvard.edu/). We run the “recon-all” processing stream with default parameters, including the following steps: first transformation to Talaraich space, intensity inhomogeneity correction, bias field correction (Sled et al., 1998), and skull-stripping (Ségonne et al., 2004). White matter (WM) was separated from everything else and the volume within the created WM–gray matter boundary was filled. After this, the surfaces were tessellated and smoothed. When these preprocessing steps were completed, the surfaces were inflated (Fischl et al., 1999) and registered to a spherical atlas.

Automatically segmented images were viewed and edited with the Freeview in line with the procedures recommended by the FreeSurfer instructions, with the addition of the Desikan–Killiany atlas that allowed us to correctly identify the areas where errors were found. Images with excess motion artefact or large unsegmented regions were excluded. The images that passed the initial quality check were then manually edited by two research staff members (authors ES and EPP, supervised by JJT) and after that the automated segmentation process was ran again as suggested by FreeSurfer instructions. The images were then inspected again for errors according to the FinnBrain quality control protocol, which is comprehensively presented in our recent article (Pulli et al., 2021).

The Freesurfer pipeline divides the cortex to 33 regions bilaterally. However, instead of analysing each region specifically, we were interested in the gross cortical anatomy and the regions of interests (ROIs) were determined for volumes and SA’s as follows: total, left and right hemispheres, and lobe division bilaterally to four main lobes (frontal, temporal, parietal and occipital) according to the Freesurfer instructions (Supplementary Table 1). We included all the MRI data that passed the FinnBrain quality control protocol. Specifically, we did not exclude participants if they had minor issues in a single or few ROIs within the lobar measures as volumes and SA’s in specific excluded regions are relatively small and are not likely to affect the measures of the main lobes and hemispheres (we also confirmed that typical errors in borders and cortical labelling were located inside main lobes instead of areas between lobes or between hemispheres). The ROI selection was justified by the notion that prior work (Jha et al., 2019) reported associations that reflected gross anatomy in the main results. In addition, expanding the approach to smaller ROI’s would have aggravated the multiple comparison issues, and we did not pursue such analyses in the current study.

### Statistical analysis

A total of 170 subjects’ MR images passed the inclusion criteria and the FinnBrain quality control protocol (Pulli et al., 2021) and were selected for the statistical analyses. Brain variables included cortical volumes and surface area for the following regions: total cortex, both cortical hemispheres, and the aforementioned four main lobes bilaterally. The refence article by Jha et al. (2019) was set as a basis for selecting the variables. After careful consideration and matching of variables, 16 demographic variables were included in the analyses. Categorial variables included: sex, NICU admission, mode of delivery, maternal and paternal education level, maternal smoking during pregnancy, diagnosis of gestational diabetes during gestation and the use of SSRI/SNRI medication during pregnancy. Continuous variables included: birth weight, gestational age at birth, postnatal age at MRI, ponderal index at MRI, maternal age at child's birth, paternal age at child's birth, maternal BMI before gestation and 5 min Apgar score. To aid interpretation of the results we have grouped the variables into three groups: 1) Fixed family factors and child features at scanning: child sex, maternal and paternal education level, maternal age at child's birth, paternal age at child's birth, postnatal age at MRI and ponderal index at MRI, 2) Incidental pregnancy and delivery related factors: mode of delivery, child birth weight and gestational age at birth as well as NICU admission and 5 min Apgar score, 3) Maternal prenatal health features: maternal pre-pregnancy BMI, maternal smoking during pregnancy, diagnosis of gestational diabetes and the use of SSRI/SNRI medication during pregnancy The following variables were considered too unreliable or otherwise suboptimal in our questionnaire data, and were excluded: maternal and paternal psychiatric history, household income, gestation number, and number of siblings. The descriptive statistics of the demographics and brain variables are presented in the Tables 1, 2 and 3.

**Table 1:** Descriptive statistics for brain variables

Surface area (mm^2^)	*N*	Mean	SD	Min	Max
	170				
Total		180,755,07	15,481,36	148,666	226,766
Left hemisphere		89,982,64	7627,79	74,749	113,230
Right hemisphere		90,772,43	7924,54	73,917	115,308
Left frontal		33,440,15	3015,67	26,272	43,656
Right frontal		33,855,83	3244,34	26,152	48,106
Left temporal		17,296,24	1544,58	13,980	21,233
Right temporal		16,907,72	1616,30	13,213	20,880
Left parietal		27,300,94	2794,00	20,793	36,058
Right parietal		27,610,06	2844,47	21,037	38,824
Left occipital		11,945,31	1286,50	8981	15,188
Right occipital		12,398,82	1414,54	8725	16,626
Volumes (mm^3^)	*N*	Mean	SD	Min	Max
	170				
Total		606,452,21	46,159,53	497,420	736,409
Left hemisphere		302,249,82	22,672,50	250,548	367,289
Right hemisphere		304,202,39	23,703,97	246,872	369,120
Left frontal		118,277,27	9006,91	91,088	139,497
Right frontal		118,764,95	9571,09	87,395	150,397
Left temporal		66,867,79	5425,63	55,329	80,265
Right temporal		65,409,89	5921,49	53,409	79,327
Left parietal		86,491,25	8331,19	69,212	109,634
Right parietal		87,297,71	8285,69	70,824	116,491
Left occipital		30,613,5	3465,59	22,948	40,722
Right occipital		32,729,84	3867,42	23,970	43,800

**Table 2:** Descriptive statistics for categorical demographic and obstetric history variables

Categorical variables		*N*	%
Sex		170	
	Male	93	54,7
	Female	77	45,4
NICU admission		169	
	Yes	23	13,6
	No	146	86,4
	Missing	1	
Mode of delivery		169	
	Vaginal	138	81,7
	C-section	31	18,3
	Missing	1	
Maternal education*		164	
	Low&Mid	83	50,6
	High	81	49,4
	Missing	6	
Paternal education*		111	
	Low&Mid	74	66,7
	High	37	33,3
	Missing	59	
Maternal smoking during gestation**		170	
	Yes	11	6,5
	No	159	93,5
Diagnosis of gestation diabetes		169	
	Yes	20	11,8
	No	149	88,2
	Missing	1	
SSRI/SNRI medication during gestation		155	
	Yes	6	3,9
	No	149	96,1
	Missing	15	
Maternal ethnicity	Finnish	165	97,1
	Other	5	2,9

*NICU* Neonatal intensive care unit; *SSRI/SNRI* Serotonin and norepinephrine reuptake inhibitors

^*^Educational levels: Low&Mid = Upper secondary school, vocational school or lower & University of applied sciences, High = University, **Maternal smoking during gestation = a combination variable of maternal smoking during early and late pregnancy

**Table 3:** Descriptive statistics for continous demographic and obstetric history variables

Continous variables	Missing	*N*	Mean	SD	Min	Max
Birth weight (g)	0	170	3550.55	477.07	2450.00	4980.00
Gestational age at birth (weeks)	0	170	39.80	1.50	35.14	42.29
Postanatal age at MRI (years)	0	170	5.40	0.13	5.09	5.79
Ponderal Index at MRI	0	170	14.10	1.20	11.21	17.63
Maternal age at child's birth (years)	0	170	30.55	4.74	19.00	41.00
Paternal age at child's birth (years)	51	119	31.88	5.14	20.00	45.00
Maternal BMI before gestation	1	169	24.27	4.33	17.48	41.95
5 min Apgar score	0	170	9.10	0.69	4	10

*BMI* Body mass index; *MRI* Magnetic resonance imaging

Statistical analyses were conducted using IBM SPSS Statistics 27.0. (IBM Corp., Armonk, NY, USA). The MRI data was confirmed to be normally distributed using JASP Statistics 0.14.1. (https://jasp-stats.org/), based on visual assessment, skewness, kurtosis and Shapiro–wilk p values (Supplementary Fig. 1 and Supplementary Table 2). Correlation matrices with Pearson correlation were created for cortical volumes and surface areas with JASP Statistics. Lateralization calculations for hemispheres and lobes were carried out with JASP statistics for descriptive purposes. Lateralization indices were also confirmed to be normally distributed based on the same criteria as the initial MRI data (Supplementary Fig. 2, Supplementary Table 2).

Linear regression models were carried out using each brain variable separately as a dependent variable and the group of 16 demographics as independent variables. Stepwise linear regression models were applied. This study was exploratory in nature and therefore we decided (a priori) not to perform formal corrections for multiple comparisons and report raw p values throughout the manuscript. The Bonferroni corrected p value at alpha level 0.05 over the 22 comparisons over the 11 regression models × 2 brain measures (SA, volumes) was p < 0.002.

## Results

### Correlation and lateralization

**Fig. 1:**
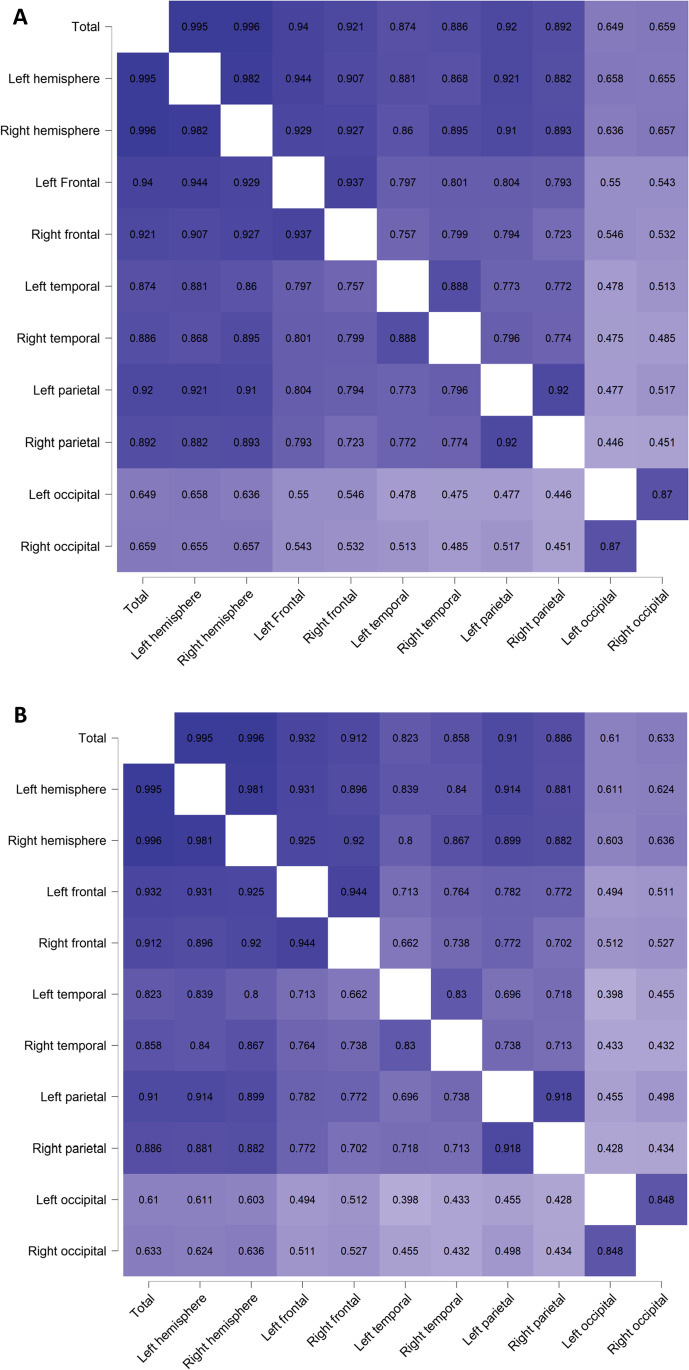
**A** Colour-coded correlation matrix of the cortical surface areas (**B**) Colour-coded correlation matrix of the cortical volumes

The total SA correlated significantly positively with hemispheric lobular SA’s (rs range = 0.649- 0.940) (Fig. 1A, Supplementary Table 3A). The SA correlations between occipital lobes and other ROI’s were remarkably weaker (rs range for the left occipital lobe = 0.44- 0.658 and for the right occipital lobe = 0.451- 0.659). Similar trends were quantified also in the cortical volumes (Fig. 1B, Supplementary Table 3B), with total volume correlating positively with hemispheric lobular volumes (rs range = 0.610- 0.932) and weaker correlations between occipital lobes and other ROI’s (rs range for the left occipital lobe = 0.398- 0.611 and for the right occipital lobe = 0.432- 0.636). Large interindividual variation in cortical anatomy (both volumes and SA) among 5-year-olds was observed.

**Fig. 2:**
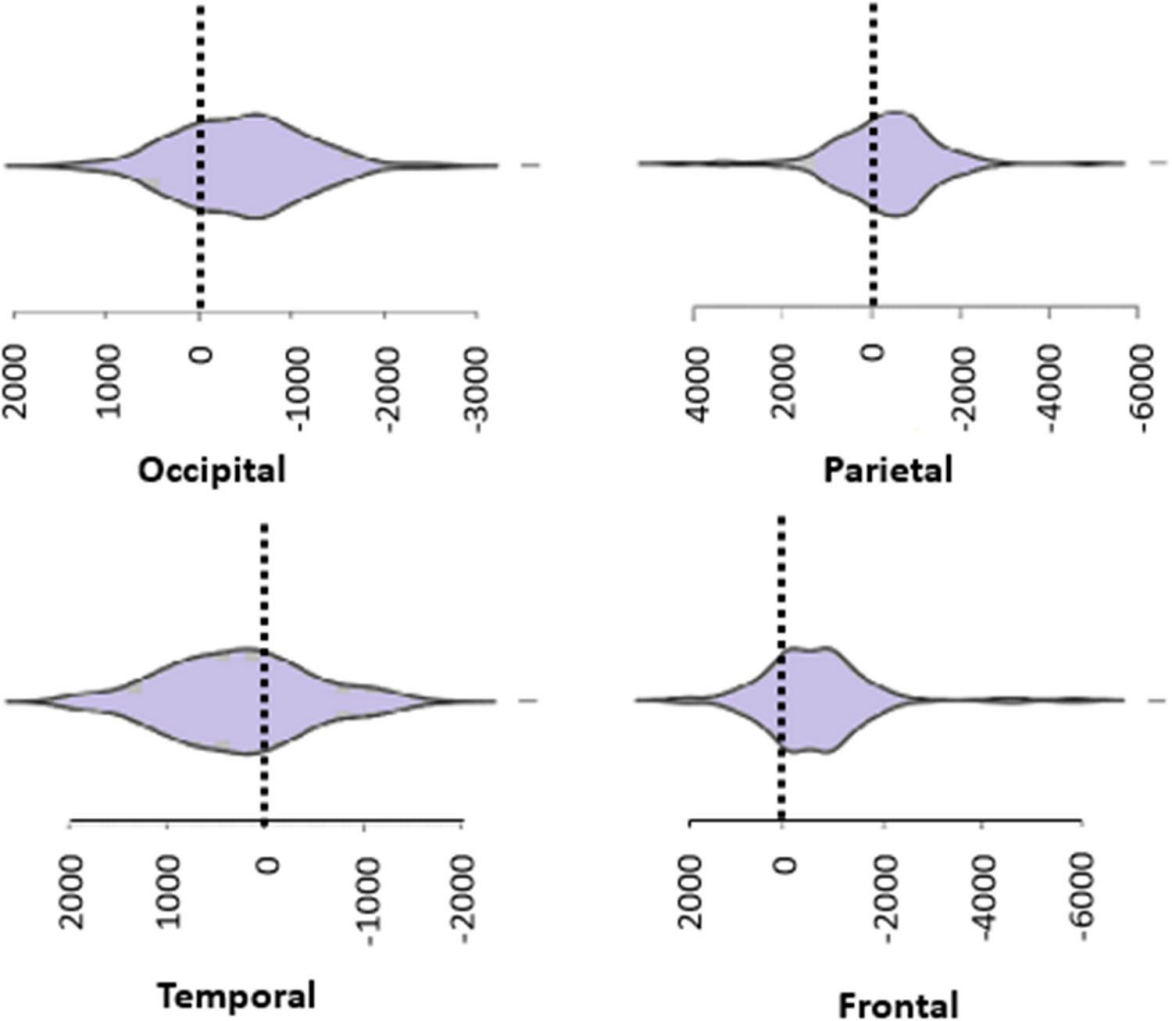
Violin plots of the lateralization of the cortical surface areas. The figure represents lateralization indexes (mm^2^), which is the difference between left and right cortical region

**Fig. 3:**
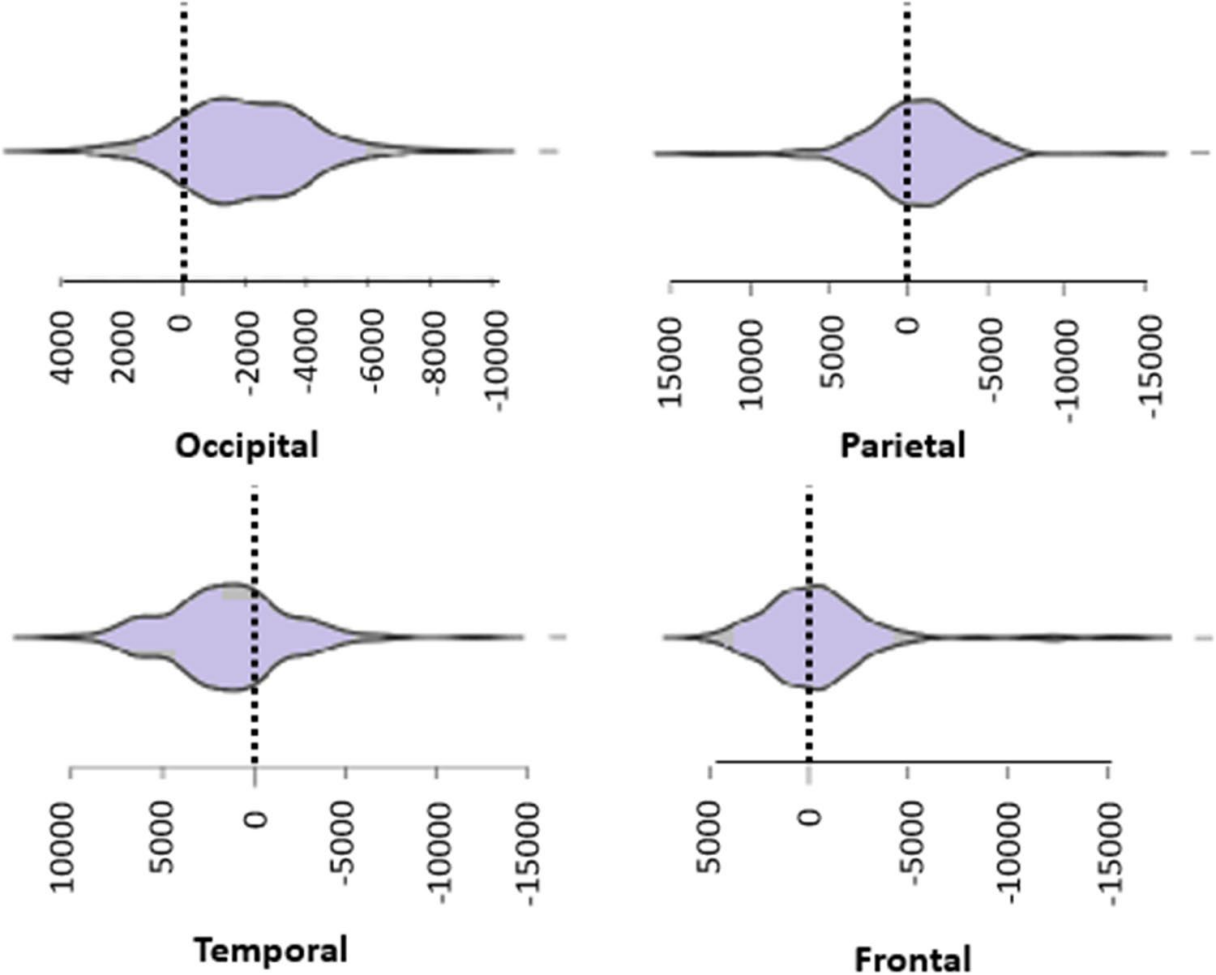
Violin plots of the lateralization of the cortical volumes. The figure represents lateralization indexes (mm^3^), which is the difference between left and right cortical region

The cortical SA’s and volumes were right lateralized except for the temporal lobes that had greater volumetric and SA values on the left side (Figs. 2 and 3). However, the degree of lateralization (the difference between hemispheres or lobes of interest divided by the total volume or SA) was relatively small in general and there was a considerable interindividual variation based on standard deviations (Supplementary Table 4).

### Surface area

The result of the regression model for total cortical surface area is presented in the Table 4 and the regression models for ROI’s in the supplementary Table 5. The reported predictors survived the threshold of p-value < 0.05. However, the only predictor that survived the Bonferroni correction at p < 0.002 was sex (with the exception of both occipital lobes). Sex was a significant predictor for SA throughout the cortex bilaterally (p < 0.001, except in both occipital lobes (p = 0.010)), with males having 8.5% larger total SA on average (M = 187 665, SD = 13 881) than females (M = 172 409, SD = 13 065). NICU admission predicted larger SA in total (p = 0.046), both hemispheres (left p = 0.049, right p = 0.020) and in the left parietal lobe (p = 0.010). Maternal smoking during pregnancy predicted smaller SA values in the right hemisphere (p = 0.040) and particularly, in the right temporal (p = 0.043), right parietal (p = 0.005) and left parietal (p = 0.039) lobes. In addition, two predictors were found for the right parietal lobe, with maternal BMI before gestation associating with larger SA (p = 0.071) and higher 5 min Apgar scores associating with smaller SA (p = 0.010).

**Table 4:** Regression model for total cortical surface area

Region of interest	Predictors	Standardized Coefficients		
		Beta	t	p
Total surface area	ΔR2 = 0.242			
	Intercept*		108.123	< 0.001
	Sex**	-0.463	-5.327	< 0.001
	NICU admission***	0.175	2.020	0.046
	Excluded predictors	Beta In	t	p
	Ponderal Index at MRI	0,111	1,284	0,202
	Gestational age at birth	-0,049	-0,557	0,579
	Maternal age at child's birth	0,096	1,093	0,277
	Paternal age at child's birth	0,068	0,779	0,438
	Maternal BMI before gestation	0,077	0,87	0,386
	Birth weight	0,026	0,3	0,765
	APGAR 5 min	-0,113	-1,171	0,244
	SSRI/SNRI medication during gestation	0,06	0,695	0,489
	Diagnosis of gestation diabetes	-0,013	-0,142	0,887
	Postanatal age at MRI	0,091	1,035	0,303
	Maternal smoking during gestation	-0,165	-1,904	0,06
	Maternal education	0,04	0,462	0,645
	Paternal education	-0,051	-0,582	0,562
	Mode of delivery	0,058	0,662	0,509

*BMI* Body mass index; *MRI* Magnetic resonance imaging; *NICU* Neonatal intensive care unit; *SSRI/SNRI* Serotonin and norepinephrine reuptake inhibitors

^*^Unstandardized Coefficients: B = 186,097.140, Std.Error = 1721.157

^**^Unstandardized Coefficients: B = -13,180.327, Std.Error = 2474.319

^***^Unstandardized Coefficients: B = 8766.857, Std.Error = 4340.761

### Volumes

The result of the regression model for total cortical volume is presented in the Table 5 and the regression models for ROI’s in the supplementary Table 6. The reported predictors survived the threshold of p-value under < 0.05. However, the only predictor that survived the Bonferroni correction at p < 0.002 was sex (with the exception of both temporal lobes). Sex was a significant predictor for volumes throughout the cortex bilaterally bilaterally (p < 0.001, except in both temporal lobes (left p = 0.048, right p = 0.009)), with males having 6.3% larger volumes on average (M = 623 594, SD = 41 419) than females (M = 585 748, SD = 43 212). Higher maternal educational level predicted larger volumes in total (p = 0.021), in the left hemisphere (p = 0.016), left frontal (p = 0.029), left temporal (p = 0.020), left parietal (p = 0.002), right temporal (p = 0.014) and in the right parietal lobes (p = 0.019). Higher maternal age predicted larger volumes in the right hemisphere (p = 0.018) and particularly, in the right parietal lobe (p = 0.006).

**Table 5:** Regression models for total cortical volume

Region of interest	Predictor	Standardized coefficients		
		Beta	t	p
Total	ΔR2 = 0.176			
	Intercept*		45.553	< 0.001
	Sex**	-0.369	-4.074	< 0.001
	Maternal education***	0.212	2.340	0.021
	Excluded predictors	Beta In	t	p
	Ponderal Index at MRI	0,089	0,983	0,328
	Gestational age at birth	-0,079	-0,859	0,392
	Maternal age at child's birth	0,169	1,863	0,065
	Paternal age at child's birth	0,114	1,221	0,225
	Maternal BMI before gestation	0,048	0,533	0,596
	Birth weight	-0,011	-0,117	0,907
	APGAR 5 min	-0,052	-0,574	0,567
	NICU admission	0,109	1,205	0,231
	SSRI/SNRI medication during gestation	0,039	0,428	0,669
	Diagnosis of gestation diabetes	0,026	0,291	0,771
	Postanatal age at MRI	0,100	1,108	0,270
	Maternal smoking during gestation	-0,112	-1,23	0,221
	Paternal education	-0,117	-1,21	0,229
	Mode of delivery	0,093	1,024	0,308

*BMI* Body mass index; *MRI* Magnetic resonance imaging; *NICU* Neonatal intensive care unit; *SSRI/SNRI* Serotonin and norepinephrine reuptake inhibitors

^*^Unstandardized Coefficients: B = 594,860.313, Std.error = 13,058.506

^**^Unstandardized Coefficients: B = -31,553.852, Std.error = 7745.375

^***^Unstandardized Coefficients: B = 18,052.615, Std.error = 7713.996

## Discussion

In the current study, we quantified how 16 family demographics and pre-/perinatal factors associate with cortical SA’s and volumes at the age of 5 years. For further discussion we grouped the variables into three groups: 1) Fixed family factors and child characteristics at scanning: child’s sex, maternal and paternal education level, maternal age at child's birth, paternal age at child's birth, postnatal age at MRI and ponderal index at MRI, 2) Incidental pregnancy and delivery related factors: mode of delivery, child birth weight and gestational age at birth as well as NICU admission and 5 min Apgar score, 3) Maternal prenatal health features: maternal pre-pregnancy BMI, maternal smoking during pregnancy, diagnosis of gestational diabetes and the use of SSRI/SNRI medication during pregnancy. Specifically, we compared the results to the ones in previous newborn studies to evaluate whether these specific factors could have possible programming effects and on the other hand, quantify the effects of environmental factors on early cortical development.

### Fixed family factors

Expectedly, child’s sex was a significant predictor for both cortical volumes and SA’s, with males having larger cortex than females. These results of absolute cortical metrics were significant globally, with the exception of occipital lobes. Larger absolute cortical volumes and SA’s are reported in also in previous newborn studies, with minor regional variation (Gilmore et al., 2007; Lehtola et al., 2019). Further, greater cortical volumes and SA’s in males have been reported in toddlers, and the difference seems to last throughout the childhood and early adulthood (Remer et al., 2017; Wilke et al., 2007). Of the studied cortical metrics, cortical thickness is reported to show weaker sexual dimorphism than volumes and SA (Wierenga et al., 2014). In addition to child’s sex, a few parental factors associated with cortical volumes, although the associations did not survive Bonferroni correction for significance.

Higher maternal education level during gestation correlated positively with total cortical volume, in the left hemisphere and partially in specific lobes bilaterally. Accordingly, maternal education has been reported to correlate positively with cortical volumes in newborns (Knickmeyer et al., 2017). Higher maternal age at delivery correlated positively with cortical volumes in the right hemisphere. This is interesting, since maternal age is a constant factor that is likely to contribute the child’s development throughout the childhood. In our study, the mean maternal age at the time of childbirth was 30.6 years (SD = 4.7 years), which is comparable to the previous work by Jha et al. (2019) (mean = 29.9, SD = 5.6 years). Previous clinical research has shown that advanced maternal age associates with reduced fertility and increased risk of offspring health problems (Jolly et al., 2000; Nassar & Usta, 2009). These conclusions of negative outcomes rely on physiological mechanisms and become more apparent at the maternal age over 35 years (Liu et al., 2011). On the other hand, epidemiologic studies have proposed that advanced maternal age (ranging from 25 to 35 years) could have positive outcome to child’s health in terms of self-rated health status, height, obesity, diagnosed conditions and mortality (Myrskylä & Fenelon, 2012). These results are likely to be partly explained by maternal SES (or other correlate of maternal age and education), which is also strongly associated with positive outcome to child’s health profile (Erola et al., 2016). Other environmental factors naturally co-influence the child’s health, too.

In comparison with the findings from the previous work by Jha et al. (2019), parental factors were not detected in newborns, whereas delivery related factors (child’s sex, birth weight, postnatal age at MRI and gestational age at birth) associated with the newborn SA. Hence, apart from child’s sex, predictors that modulated SA in newborns were different than predictors that modulated SA in 5-year-olds.

### Maternal prenatal health profile and delivery related factors

NICU admission and maternal BMI before gestation correlated positively with cortical SA, whereas 5 min Apgar score and maternal smoking during pregancy correlated negatively with cortical SA at the age of 5 years. These findings did not survive the Bonferroni correction, though. In comparison with these findings to the ones in the previous work by Jha et al. (2019), maternal health profile during gestation and incidental delivery related features did not associate with cortical SA in the newborns. The previous study found also positive correlations between cortical SA and birth weight in newborns, but this association was not detected in our cohort. Nevertheless, it is suggested that birth weight is a prenatal factor that could have long-lasting influences in the cortical morphology (Gilmore et al., 2020; Walhovd et al., 2016).

Maternal smoking during pregnancy predicted smaller SA values comprehensively in the right hemisphere, with the exception of the right frontal lobe showing no significant associations. These results support the findings from previous studies, where pre/perinatal smoking exposure predicts smaller SA, CT and brain volumes (el Marroun et al., 2016; Liu et al., 2013; Salminen et al., 2019), likely due to the growth restriction caused by nicotine-induced hypoxemia (Lambers & Clark, 1996).

### Patterns of lateralization

The lateralization of the cortical anatomy was right sided, with only the temporal lobes showing greater values on the left hemisphere. However, the degree of lateralization was modest in general. These findings differed remarkably compared to what has been reported among newborns (Lehtola et al., 2019), where rightward asymmetry was detected in temporal lobe and leftward asymmetry in occipital and parietal lobes. While previous research has commonly reported rightward asymmetry of the brain from the childhood to early adulthood (Dean et al., 2018; Tanaka et al., 2013), leftward asymmetry has also been detected in infants (Gilmore et al., 2007). The age-related trends of the cortical asymmetry form a potentially interesting phenotype, e.g. for language related studies.

### Strengths and limitations

The strengths of this study include relatively large sample size with prospective design. The MRI data is analysed thoroughly with quality protocols carried out for every subject and hence, we provide state of art imaging data. Most demographics were collected from national birth registry that is built from records of clinical staff, which makes the variables reliable and standardized. Some variables were missing in our study compared to the previous work by Jha et al. (2019), but generally the environmental and obstetric variables matched satisfactorily.

There were a few limitations, too. Part of the demographics were obtained from questionnaires from mothers during pregnancy (including paternal information), which creates minor but important reliability considerations. The 5-year time gap between data collection and imaging sessions was intended as per study design as we were specifically interested in whether a set of demographics that have been shown to be important determinants of infant brain structure have similar associations later in development. Full longitudinal data would be optimal for this purpose and such studies are warranted in the light of our results. In addition, some variables included a modest sample size both absolutely and in relation to the whole data (e.g. maternal smoking and the use of medication during pregnancy). Even though the demographics matched satisfactorily compared to the previous work (Jha et al., 2019), the brain variables differed partly between the studies, as we analyzed cortical SA and volume, whereas Jha et al. studied CT and SA, which is in our view age-appropriate. Most of these limitations can likely be addressed in data sets such as the developing and baby Human Connectome Project (Eggebrecht et al., 2018; Fenchel et al., 2020).

## Conclusions

In summary, we aimed to add knowledge about the influence of environmental factors and family demographics on brain cortical SA and volume in healthy 5-year-olds. We wanted to inspect whether our current findings are in line with similar studies carried out with newborns in order to evaluate possible age-related and developmental differences in factors explaining the variance in brain cortical structure. We found that, apart from child sex, the predictors of cortical volume and SA that are typically detected in infant MRI studies were not identified here at the child age of 5 years. This could be due to variation in methodology or study population characteristics but can also be interpreted as preliminary evidence on early childhood postnatal environmental factors influencing cortical development over prenatal factors as children grow up. Overall, despite the large variable pool, only few of the candidate variables were found to predict cortical structural features. Hence, it is worth mentioning, that the aim of this study was not to find the best predictive model for 5-year-olds, but to explore similarities and differences of the variables in comparison with earlier infant studies. These findings suggest that although closely related to each other, cortical SA and volumes have different developmental patterns with differential associations with observed variables. Overall, the effects of prenatal and early life variables on cortical development are not standardised in the current literature, and hence it is important to report these factors if they are available. In the future, researchers will likely benefit from including similar environmental, parental and delivery related variables to sensitivity analyses in studies on cortical anatomy between 0–5 years of life either by carrying out longitudinal studies or obtaining data retrospectively.

## Supplementary Information

Below is the link to the electronic supplementary material.

Supplementary file1 (DOCX 840 KB)

## Data Availability

The data can not be made openly available due to restrictions by national law and local ethical permissions. Data sharing is possible via formal material transfer agreements for which interested investigators should contact the authors.

## References

[CR1] Ball WS, Byars AW, Schapiro M, Bommer W, Carr A, German A, O’Neill J (2012). Total and regional brain volumes in a population-based normative sample from 4 to 18 years: The NIH MRI study of normal brain development. Cerebral Cortex.

[CR2] Copeland A, Silver E, Korja R, Lehtola S, Merisaari H, Saukko E, Sinisalo S, Saunavaara J, Tuulari JJ (2021). Infant and child MRI: A review of scanning procedures. Frontiers in Neuroscience.

[CR3] Dean DC, Planalp EM, Wooten W, Schmidt CK, Kecskemeti SR, Frye C, Davidson RJ (2018). Investigation of brain structure in the 1-month infant. Brain Structure and Function.

[CR4] Duan C, Hare MM, Staring M, Deligiannidis KM (2019). Examining the relationship between perinatal depression and neurodevelopment in infants and children through structural and functional neuroimaging research. International Review of Psychiatry.

[CR5] Eggebrecht, A. T., Elison, J. T., Feczko, E., Todorov, A., Wolff, J. J., Kandala, S., … Pruett, J. R. (2018). Joint attention and brain functional connectivity in infants and toddlers. *Cerebral Cortex* (March 2017), 1709–1720. 10.1093/cercor/bhw403 10.1093/cercor/bhw403 PMC5452276 28062515

[CR6] el Marroun H, Tiemeier H, Franken IHA, Jaddoe VWV, van der Lugt A, Verhulst FC, Lahey BB, White T (2016). Prenatal Cannabis and tobacco exposure in relation to brain morphology: A prospective neuroimaging study in young children. Biological Psychiatry.

[CR7] Erola J, Jalonen S, Lehti H (2016). Parental education, class and income over early life course and children’s achievement. Research in Social Stratification and Mobility.

[CR8] Fenchel D, Dimitrova R, Seidlitz J, Robinson EC, Batalle D, Hutter J, O’Muircheartaigh J (2020). Development of Microstructural and Morphological Cortical Profiles in the Neonatal Brain. Cerebral Cortex.

[CR9] Fischl B, Sereno MI, Dale AM (1999). Cortical surface-based analysis: II. Inflation, flattening, and a surface-based coordinate system. NeuroImage.

[CR10] Gilmore JH, Langworthy B, Girault JB, Fine J, Jha SC, Kim SH, Styner M (2020). Individual variation of human cortical structure is established in the first year of life. Biological Psychiatry: Cognitive Neuroscience and Neuroimaging.

[CR11] Gilmore JH, Lin W, Prastawa MW, Looney CB, Vetsa YSK, Knickmeyer RC, Gerig G (2007). Regional gray matter growth, sexual dimorphism, and cerebral asymmetry in the neonatal brain. Journal of Neuroscience.

[CR12] Hazlett HC, Poe M, Gerig G, Styner M, Chappell C, Smith RG, Piven J (2012). Early brain overgrowth in autism associated with an increase in cortical surface area before age 2. Bone.

[CR13] Jha SC, Xia K, Ahn M, Girault JB, Li G, Wang L, Knickmeyer RC (2019). Environmental influences on infant cortical thickness and surface area. Cerebral Cortex.

[CR14] Jolly M, Sebire N, Harris J, Robinson S, Regan L (2000). The risks associated with pregnancy in women aged 35 years or older. Human Reproduction.

[CR15] Karlsson L, Tolvanen M, Scheinin NM, Uusitupa H-M, Korja R, Ekholm E, Karlsson H (2018). Cohort profile: The FinnBrain birth cohort study (FinnBrain). International Journal of Epidemiology.

[CR16] Knickmeyer RC, Gouttard S, Kang C, Evans D, Smith JK, Hamer RM, Lin W, Gerig G, John H (2008). A structural MRI study of human brain development from birth to 2 years. Journal of Neuroscience.

[CR17] Kuhl, U., Friederici, A. D., Emmrich, F., Brauer, J., Wilcke, A., Neef, N., … Skeide, M. A. (2020). Early cortical surface plasticity relates to basic mathematical learning. *NeuroImage*, *204*(October 2019). 10.1016/j.neuroimage.2019.116235 10.1016/j.neuroimage.2019.116235 31586675

[CR18] Lambers DS, Clark KE (1996). The maternal and fetal physiologic effects of nicotine. Seminars in Perinatology.

[CR19] Lehtola SJ, Tuulari JJ, Karlsson L, Parkkola R, Merisaari H, Saunavaara J, Karlsson H (2019). Associations of age and sex with brain volumes and asymmetry in 2–5-week-old infants. Brain Structure and Function.

[CR20] Li G, Wang L, Shi F, Lyall AE, Ahn M, Peng Z, Shen D (2016). Cortical thickness and surface area in neonates at high risk for schizophrenia. Brain Structure and Function.

[CR21] Liu J, Lester BM, Neyzi N, Sheinkopf SJ, Gracia L, Kekatpure M, Kosofsky BE (2013). Regional brain morphometry and impulsivity in adolescents following prenatal exposure to cocaine and tobacco. JAMA Pediatrics.

[CR22] Liu Y, Zhi M, Li X (2011). Parental age and characteristics of the offspring. Ageing Research Reviews.

[CR23] Lyall AE, Shi F, Geng X, Woolson S, Li G, Wang L, Gilmore JH (2015). Dynamic development of regional cortical thickness and surface area in early childhood. Cerebral Cortex.

[CR24] Merisaari H, Tuulari JJ, Karlsson L, Scheinin NM, Parkkola R, Saunavaara J, Lähdesmäki T, Lehtola SJ, Keskinen M, Lewis JD, Evans AC, Karlsson H (2019). Test-retest reliability of Diffusion Tensor Imaging metrics in neonates. NeuroImage.

[CR25] Myrskylä, M., & Fenelon, A. (2012). Maternal age and offspring adult health: Evidence from the health and retirement study. Demography, 49(4), 1231–1257. 10.1007/s13524-012-0132-x 10.1007/s13524-012-0132-x PMC3881604 22926440

[CR26] Nassar AH, Usta IM (2009). Advanced maternal age. part II: Long-term consequences. American Journal of Perinatology.

[CR27] Pulli EP, Kumpulainen V, Kasurinen JH, Korja R, Merisaari H, Karlsson L, Tuulari JJ (2019). Prenatal exposures and infant brain: Review of magnetic resonance imaging studies and a population description analysis. Human Brain Mapping.

[CR28] Pulli, E. P., Silver, E., Kumpulainen, V., Copeland, A., Merisaari, H., Saunavaara, J., Parkkola, R., Lähdesmäki, T., Saukko, E., Nolvi, S., Kataja, E-L., Korja, R., Karlsson, L., Karlsson, H., Tuulari, J. (2021). Feasibility of FreeSurfer processing for T1-weighted brain images of 5-year- olds: semiautomated protocol of FinnBrain Neuroimaging Lab Authors. *Front. Neurosci.*10.3389/fnins.2022.874062 10.3389/fnins.2022.874062 PMC9108497 35585923

[CR29] Remer J, Croteau-Chonka E, Dean DC, D’Arpino S, Dirks H, Whiley D, Deoni SCL (2017). Quantifying cortical development in typically developing toddlers and young children, 1–6 years of age. NeuroImage.

[CR30] Salminen LE, Wilcox RR, Zhu AH, Riedel BC, Ching CRK, Rashid F, Thomopoulos SI, Saremi A, Harrison MB, Ragothaman A, Knight V, Boyle CP, Medland SE, Thompson PM, Jahanshad N (2019). Altered cortical brain structure and increased risk for disease seen decades after perinatal exposure to maternal smoking: A study of 9000 adults in the UK Biobank. Cerebral Cortex.

[CR31] Ségonne F, Dale AM, Busa E, Glessner M, Salat D, Hahn HK, Fischl B (2004). A hybrid approach to the skull stripping problem in MRI. NeuroImage.

[CR32] Sheridan MA, Sarsour K, Jutte D, D’Esposito M, Boyce WT (2012). The impact of social disparity on prefrontal function in childhood. PLoS ONE.

[CR33] Sled JG, Zijdenbos AP, Evans AC (1998). A nonparametric method for automatic correction of intensity nonuniformity in mri data. IEEE Transactions on Medical Imaging.

[CR34] Tanaka C, Matsui M, Uematsu A, Noguchi K, Miyawaki T (2013). Developmental trajectories of the fronto-temporal lobes from infancy to early adulthood in healthy individuals. Developmental Neuroscience.

[CR35] Toga AW, Thompson PM (2003). Mapping brain asymmetry. Nature Reviews Neuroscience.

[CR36] Walhovd KB, Krogsrud SK, Amlien IK, Bartsch H, Bjørnerud A, Due-Tønnessen P, Fjell AM (2016). Neurodevelopmental origins of lifespan changes in brain and cognition. Proceedings of the National Academy of Sciences of the United States of America.

[CR37] Wierenga LM, Langen M, Oranje B, Durston S (2014). Unique developmental trajectories of cortical thickness and surface area. NeuroImage.

[CR38] Wilke M, Krägeloh-Mann I, Holland SK (2007). Global and local development of gray and white matter volume in normal children and adolescents. Experimental Brain Research.

[CR39] Wu Y, Lu YC, Jacobs M, Pradhan S, Kapse K, Zhao L, Limperopoulos C (2020). Association of prenatal maternal psychological distress with fetal brain growth, metabolism, and cortical maturation. JAMA Network Open.

[CR40] Yang DYJ, Beam D, Pelphrey KA, Abdullahi S, Jou RJ (2016). Cortical morphological markers in children with autism: A structural magnetic resonance imaging study of thickness, area, volume, and gyrification. Molecular Autism.

