# Supplementary material for: Prenatal and early-life environmental factors, family demographics and cortical brain anatomy in 5-year-olds: an MRI study from FinnBrain Birth Cohort
Source: Brain Imaging Behav. 2022 Jul 22;16(5):2097–109. doi: 10.1007/s11682-022-00679-w (PMC9581828; doi:10.1007/s11682-022-00679-w)
Supplement: Supplementary file 1 — Supplementary file1 (DOCX 840 KB) [file 11682_2022_679_MOESM1_ESM.docx]

**Supplementary Table 1**. Lobe division. In the current study, The cingulate was included to the lobes according to the list. The list has been printed from FreeSurfer wiki, available at the time of writing from <https://surfer.nmr.mgh.harvard.edu/fswiki/CorticalParcellation>.


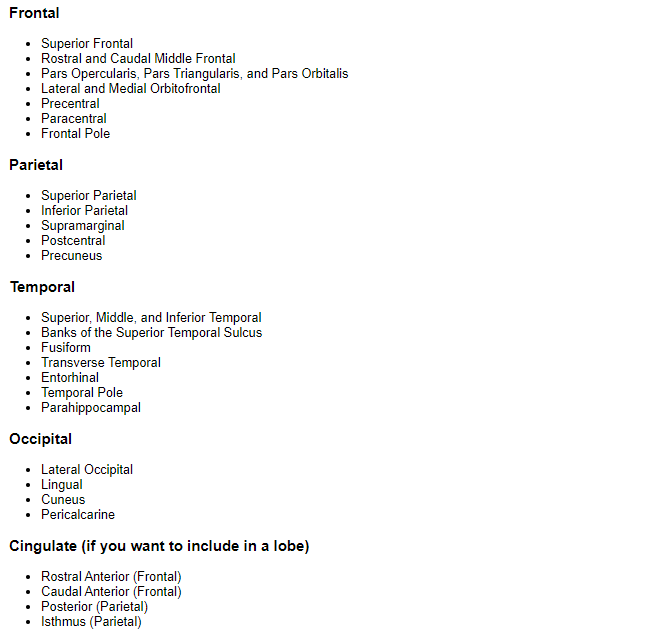


| **Supplementary Table 2**. Detailed descriptive statistics of the brain variables. | | | | | | |  |  |  |  |  |  |  |
| --- | --- | --- | --- | --- | --- | --- | --- | --- | --- | --- | --- | --- | --- |
| Region of interest | | Valid | Missing | Mean | SD | Skewness | Std. Error of Skewness | Kurtosis | Std. Error of Kurtosis | Shapiro-Wilk | p | Minimum | Maximum |
|  |  | 170 | 0 |  |  |  |  |  |  |  |  |  |  |
| Surface areas (mm²) | |  |  |  |  |  |  |  |  |  |  |  |  |
| Lh_Frontal_SA | |  |  | 33440.147 | 3015.673 | 0.162 | 0.186 | 0.096 | 0.370 | 0.994 | 0.663 | 26272.000 | 43656.000 |
| Rh_Frontal_SA | |  |  | 33855.829 | 3244.335 | 0.372 | 0.186 | 1.488 | 0.370 | 0.981 | 0.020 | 26152.000 | 48106.000 |
| Lh_Temporal_SA | |  |  | 17296.241 | 1544.579 | 0.047 | 0.186 | -0.509 | 0.370 | 0.990 | 0.302 | 13980.000 | 21233.000 |
| Rh_Temporal_SA | |  |  | 16907.724 | 1616.298 | 0.134 | 0.186 | -0.517 | 0.370 | 0.991 | 0.335 | 13213.000 | 20880.000 |
| Lh_Parietal_SA | |  |  | 27300.941 | 2793.995 | 0.309 | 0.186 | 0.077 | 0.370 | 0.991 | 0.397 | 20793.000 | 36058.000 |
| Rh_Parietal_SA | |  |  | 27610.059 | 2844.469 | 0.499 | 0.186 | 0.924 | 0.370 | 0.983 | 0.037 | 21037.000 | 38824.000 |
| Lh_Occip_SA | |  |  | 11945.312 | 1286.495 | 0.164 | 0.186 | -0.175 | 0.370 | 0.990 | 0.303 | 8981.000 | 15188.000 |
| Rh_Occip_SA | |  |  | 12398.818 | 1414.536 | 0.395 | 0.186 | 0.427 | 0.370 | 0.987 | 0.129 | 8725.000 | 16626.000 |
| Lh_SA |  |  |  | 89982.641 | 7627.787 | 0.219 | 0.186 | -0.002 | 0.370 | 0.986 | 0.082 | 74749.000 | 113230.000 |
| Rh_SA |  |  |  | 90772.429 | 7924.538 | 0.267 | 0.186 | 0.132 | 0.370 | 0.987 | 0.105 | 73917.000 | 115308.000 |
| Total_SA |  |  |  | 180755.071 | 15481.358 | 0.248 | 0.186 | 0.067 | 0.370 | 0.985 | 0.072 | 148666.000 | 226766.000 |
| Volumes (mm³) | |  |  |  |  |  |  |  |  |  |  |  |  |
| Lh_FrontalVol | |  |  | 118277.271 | 9006.911 | 0.094 | 0.186 | 0.032 | 0.370 | 0.991 | 0.391 | 91088.000 | 139497.000 |
| Rh_FrontalVol | |  |  | 118764.953 | 9571.089 | 0.197 | 0.186 | 0.819 | 0.370 | 0.990 | 0.249 | 87395.000 | 150397.000 |
| Lh_Temporal_Vol | |  |  | 66867.794 | 5425.633 | 0.134 | 0.186 | -0.583 | 0.370 | 0.986 | 0.081 | 55329.000 | 80265.000 |
| Rh_Temporal_Vol | |  |  | 65409.888 | 5921.492 | 0.208 | 0.186 | -0.482 | 0.370 | 0.985 | 0.064 | 53409.000 | 79327.000 |
| Lh_Parietal_Vol | |  |  | 86491.253 | 8331.192 | 0.320 | 0.186 | 0.024 | 0.370 | 0.985 | 0.062 | 69212.000 | 109634.000 |
| Rh_Parietal_Vol | |  |  | 87297.712 | 8285.687 | 0.477 | 0.186 | 0.697 | 0.370 | 0.977 | 0.007 | 70824.000 | 116491.000 |
| Lh_Occip_Vol | |  |  | 30613.500 | 3465.594 | 0.353 | 0.186 | 0.202 | 0.370 | 0.987 | 0.102 | 22948.000 | 40722.000 |
| Rh_Occip_Vol | |  |  | 32729.835 | 3867.415 | 0.385 | 0.186 | 0.112 | 0.370 | 0.985 | 0.058 | 23970.000 | 43800.000 |
| Lh_Vol |  |  |  | 302249.818 | 22672.504 | 0.215 | 0.186 | -0.055 | 0.370 | 0.993 | 0.557 | 250548.000 | 367289.000 |
| Rh_Vol |  |  |  | 304202.388 | 23703.967 | 0.215 | 0.186 | 0.076 | 0.370 | 0.993 | 0.530 | 246872.000 | 369120.000 |
| Total_Vol |  |  |  | 606452.206 | 46159.528 | 0.216 | 0.186 | 0.011 | 0.370 | 0.993 | 0.601 | 497420.000 | 736409.000 |
| LI_Frontal_SA | |  |  | -415.682 | 1133.870 | -1.561 | 0.186 | 5.972 | 0.370 | 0.883 | < .001 | -5801.000 | 2395.000 |
| LI_Temporal_SA | |  |  | 388.518 | 752.446 | -0.095 | 0.186 | -0.222 | 0.370 | 0.992 | 0.493 | -1535.000 | 2066.000 |
| LI_Parietal_SA | |  |  | -309.118 | 1128.542 | 0.448 | 0.186 | 3.266 | 0.370 | 0.953 | < .001 | -4853.000 | 4381.000 |
| LI_Occip_SA | |  |  | -453.506 | 699.051 | -0.102 | 0.186 | 0.125 | 0.370 | 0.996 | 0.893 | -2580.000 | 1441.000 |
| LI_SA |  |  |  | -789.788 | 1513.406 | 0.229 | 0.186 | 0.736 | 0.370 | 0.989 | 0.236 | -5549.000 | 4465.000 |
| LI_Frontal_Vol | |  |  | -487.682 | 3147.307 | -2.172 | 0.186 | 7.860 | 0.370 | 0.827 | < .001 | -16181.000 | 5297.000 |
| LI_Temporal_Vol | |  |  | 1457.906 | 3345.067 | -0.345 | 0.186 | 1.208 | 0.370 | 0.984 | 0.049 | -12092.000 | 10408.000 |
| LI_Parietal_Vol | |  |  | -806.459 | 3358.865 | 0.613 | 0.186 | 3.369 | 0.370 | 0.948 | < .001 | -13903.000 | 13193.000 |
| LI_Occip_Vol | |  |  | -2116.335 | 2056.968 | -0.210 | 0.186 | 0.559 | 0.370 | 0.991 | 0.411 | -8840.000 | 3708.000 |
| LI_Vol |  |  |  | -1952.571 | 4597.712 | -0.132 | 0.186 | 0.318 | 0.370 | 0.986 | 0.095 | -13136.000 | 12257.000 |
|  |  |  |  |  |  |  |  |  |  |  |  |  |  |
| Lh= Left hemisphere, LI= Lateralization Index, the difference between left hemisphere/lobe and right hemisphere/lobe. SA= Surface Area, Vol= Volume, SD= Standard Deviation. | | | | | | | | | | | | |  |

**Supplementary Table 3A**. Correlation matrix of the cortical surface areas


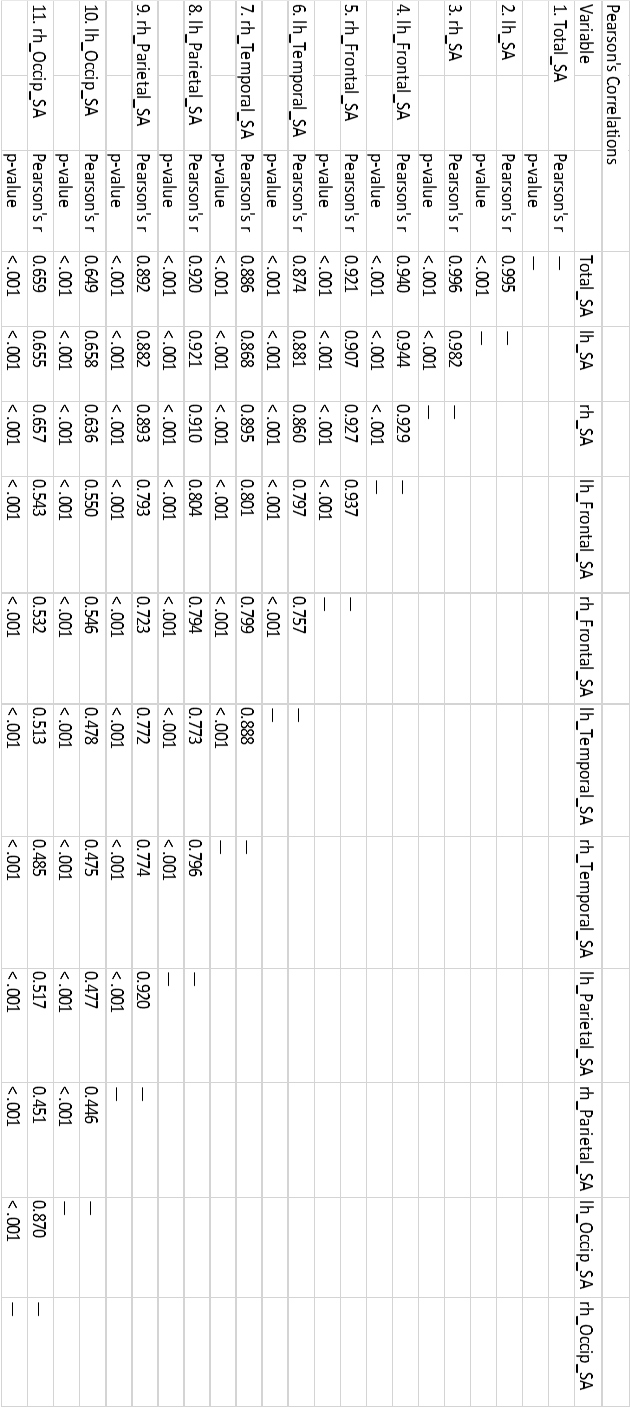


lh= left hemisphere, rh= right hemisphere, SA= Surface Area.

**Supplementary Table 3B**. Correlation matrix of the cortical volumes.

lh= left hemisphere, rh= right hemisphere

**Supplementary Table 4.** Descriptive statistics of the lateralization of the cortical volumes and surface areas

Deg= The degree of lateralization, the difference between hemispheres/lobes divided with a total cortical SA/volume). LI= Lateralization Index, the difference between left hemisphere/lobe and right hemisphere/lobe. SA= Surface Area, Vol= Volume, SD= Standard Deviation.

**Supplementary Figure 1.** Violin plots of the brain volumes and surface areas.

**lh_Frontal_SA rh_Frontal_SA**


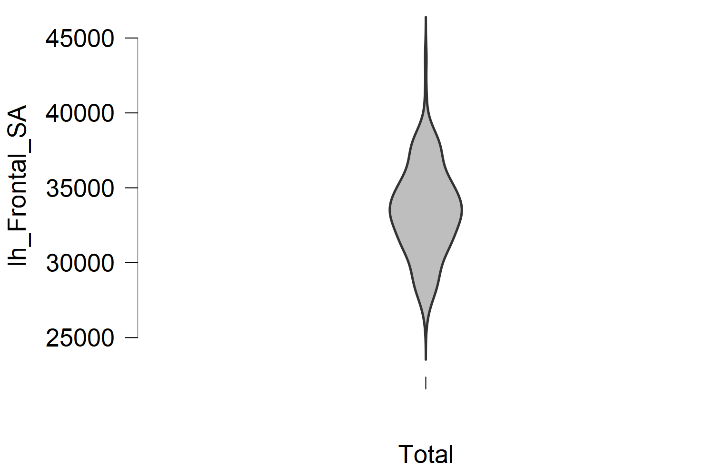

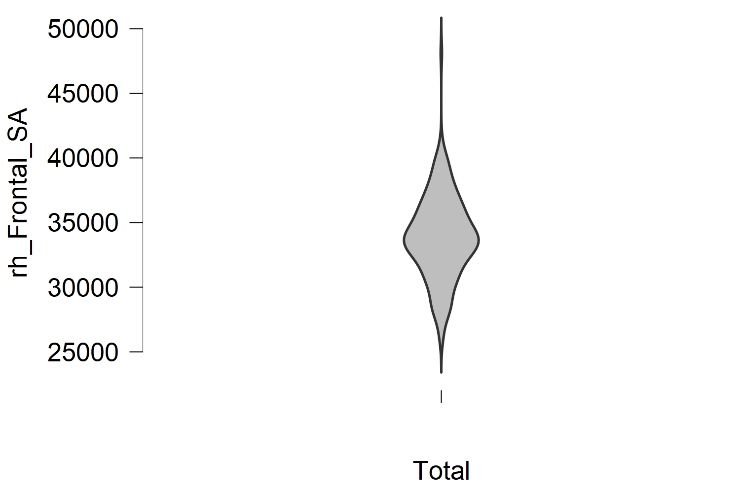


**lh_Temporal_SA rh_Temporal_SA**


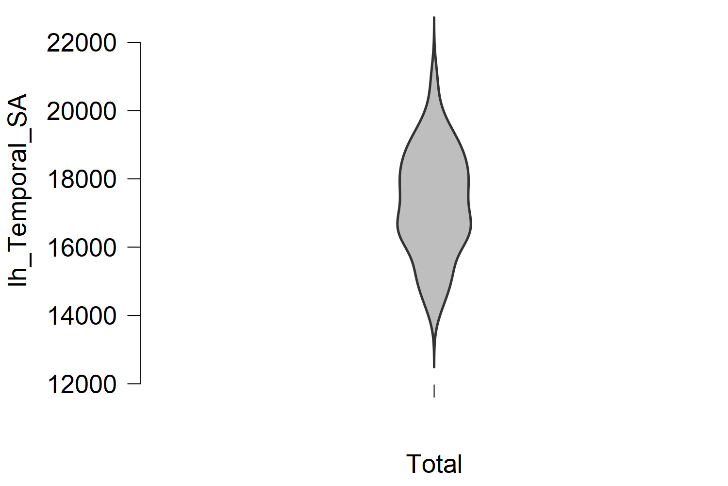

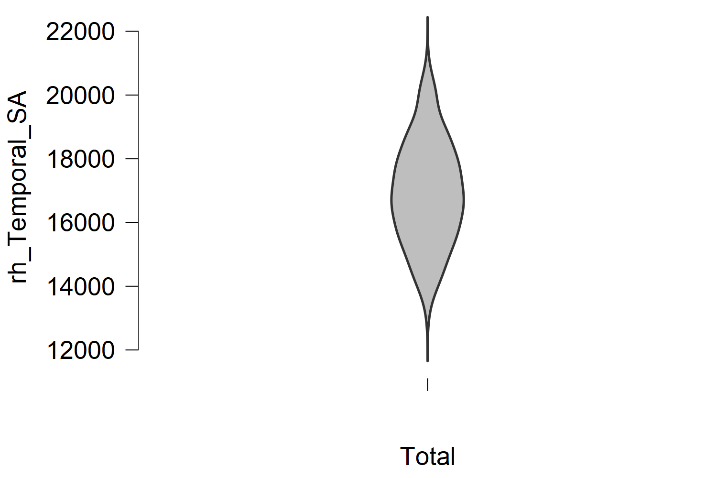


**lh_Parietal_SA rh_Parietal_SA**


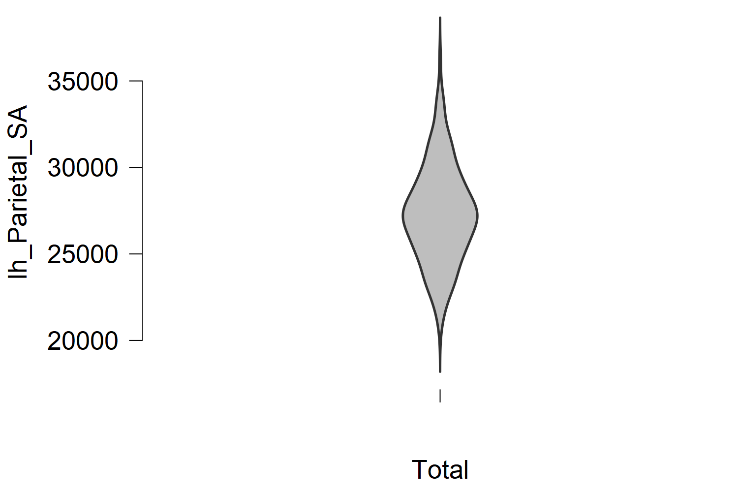

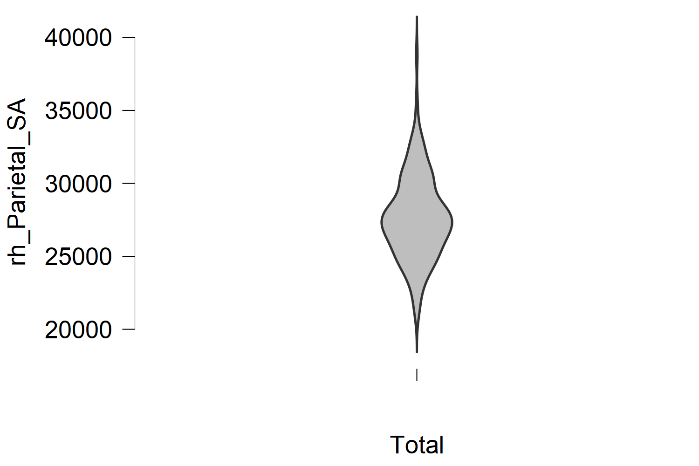


**lh_Occip_SA rh_Occip_SA**


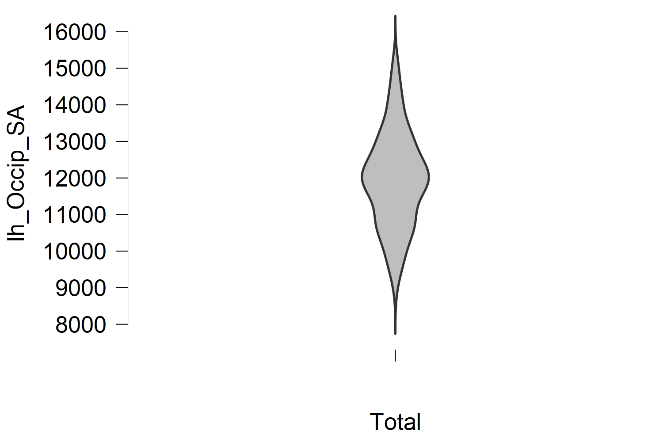

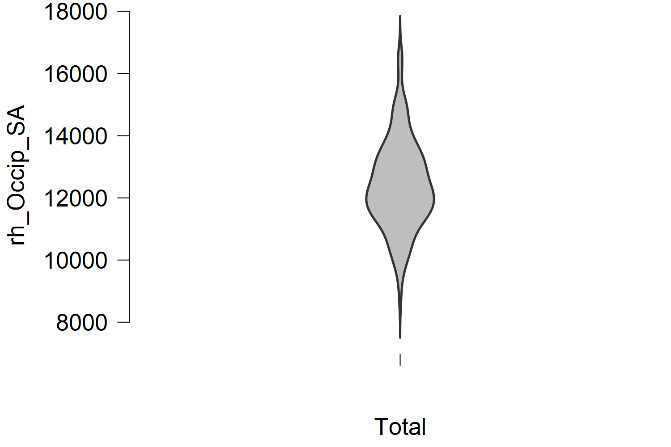


**lh_SA rh_SA**


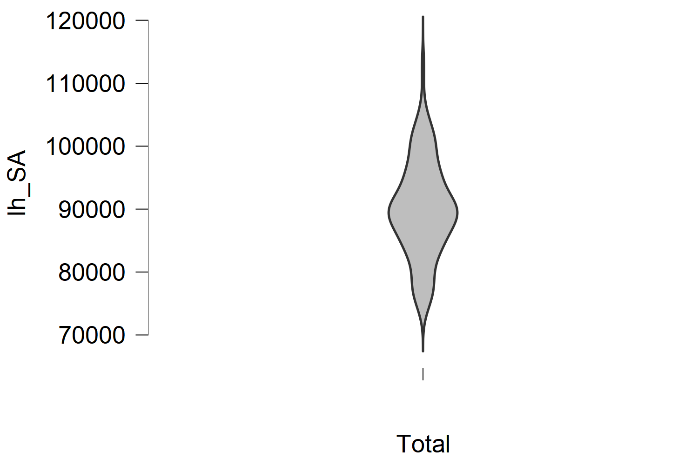

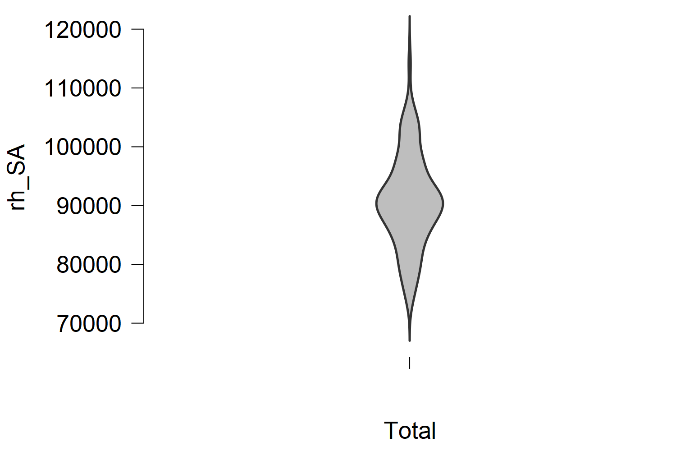


**Total_SA**


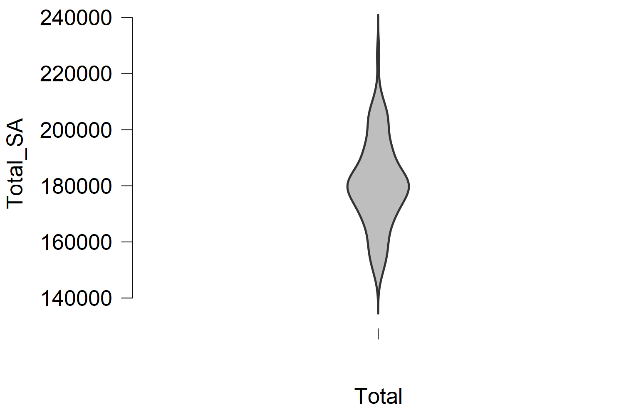


**lh_FrontalVol rh_FrontalVol**


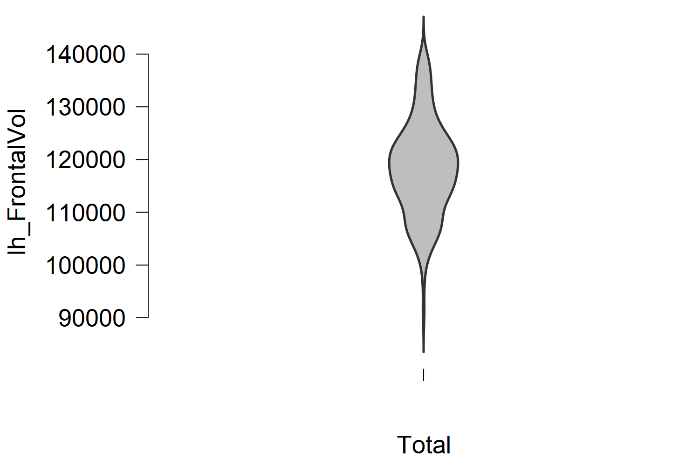

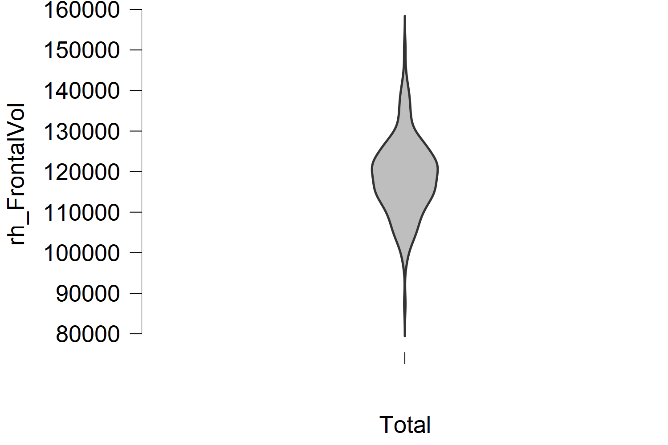


**lh_Temporal_Vol rh_Temporal_Vol**


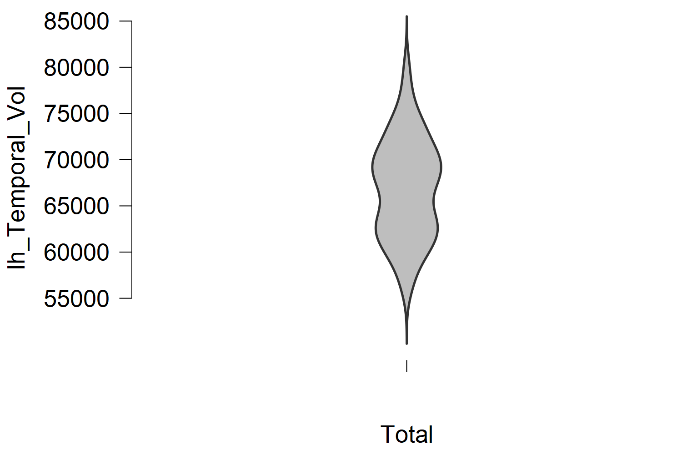

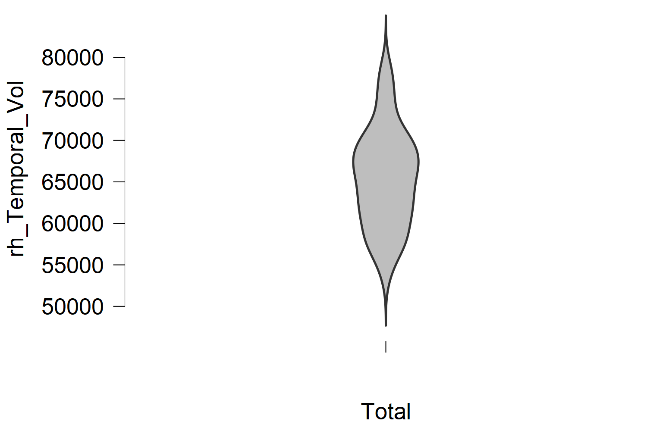


**lh_Parietal_Vol rh_Parietal_Vol**


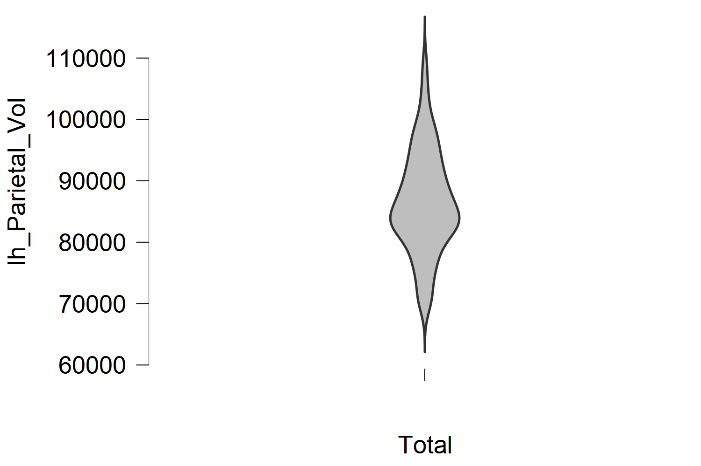

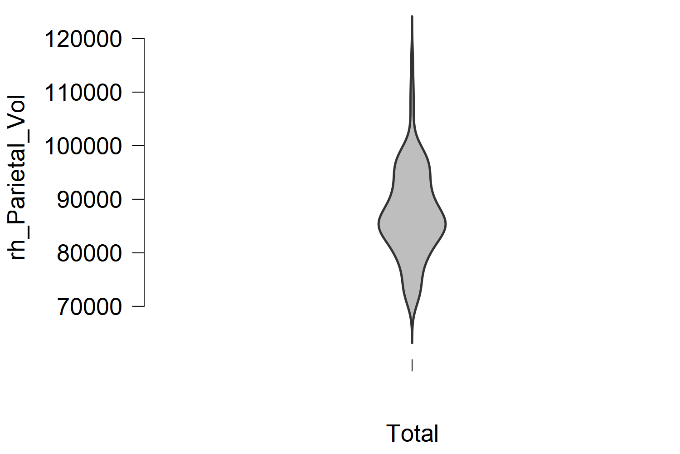


**lh_Occip_Vol rh_Occip_Vol**


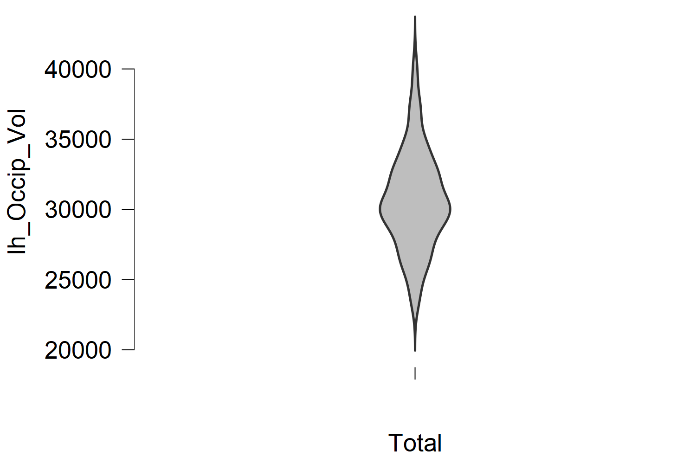

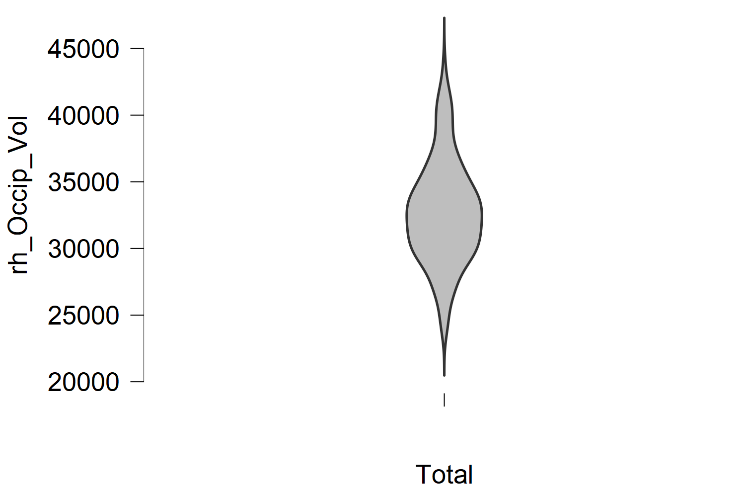


**lh_Vol rh_Vol**


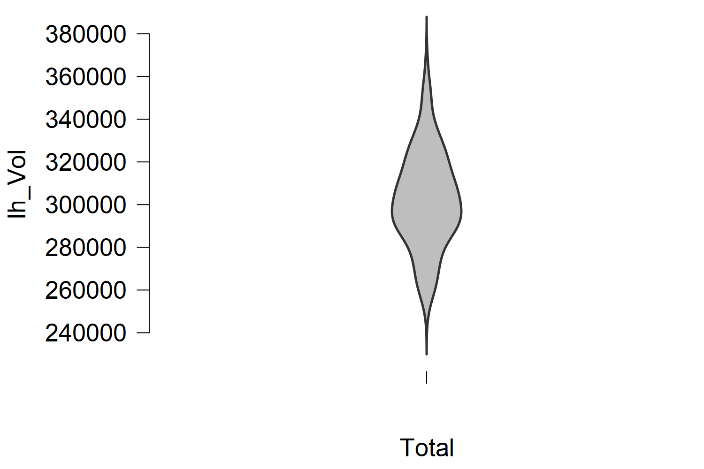

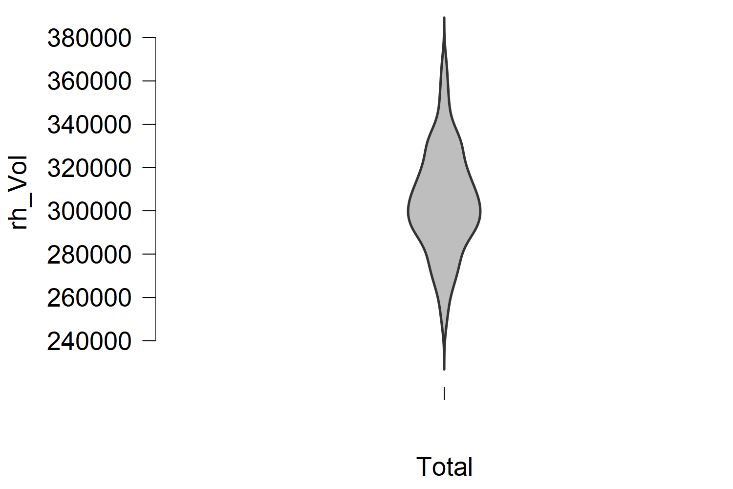


**Total_Vol**


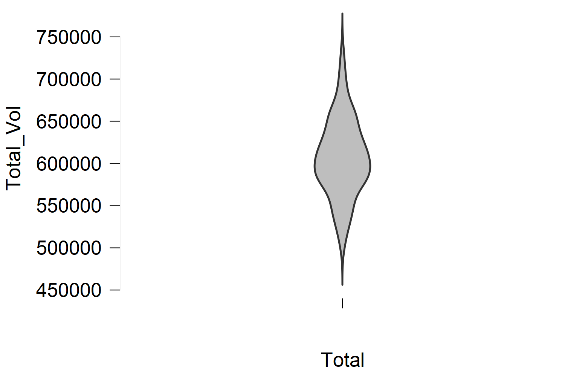


lh= left hemisphere, rh= right hemisphere, SA= Surface Area, Vol= Volumes

**Supplementary Figure 2.** Violin plots of the lateralization of the cortical volumes and surface areas.


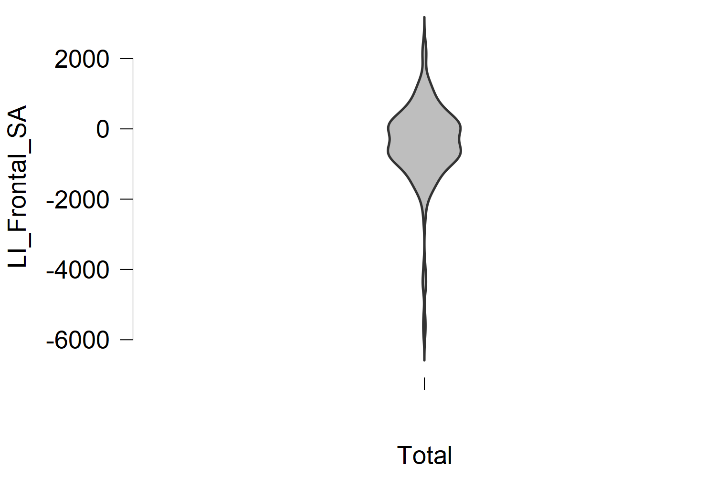

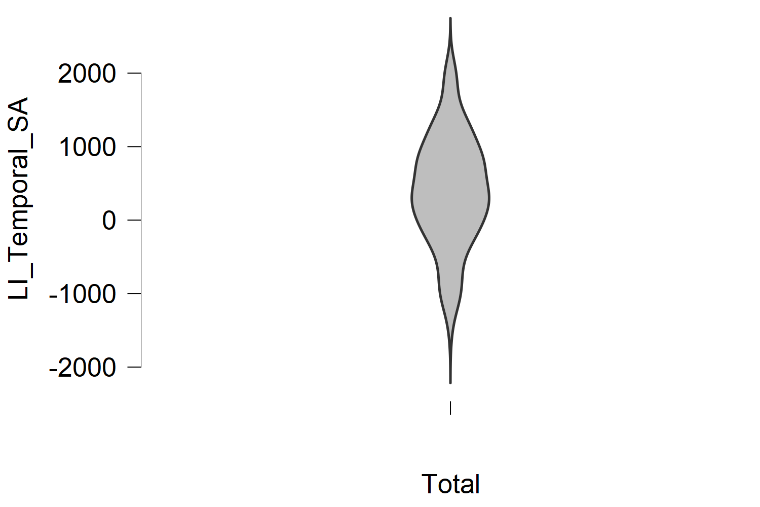


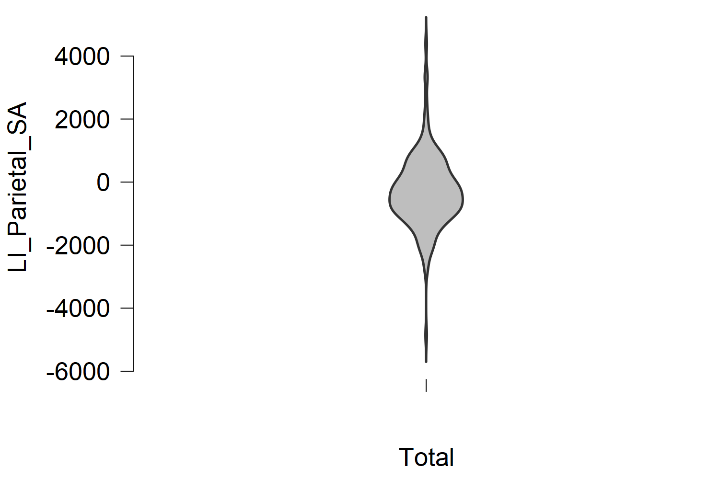

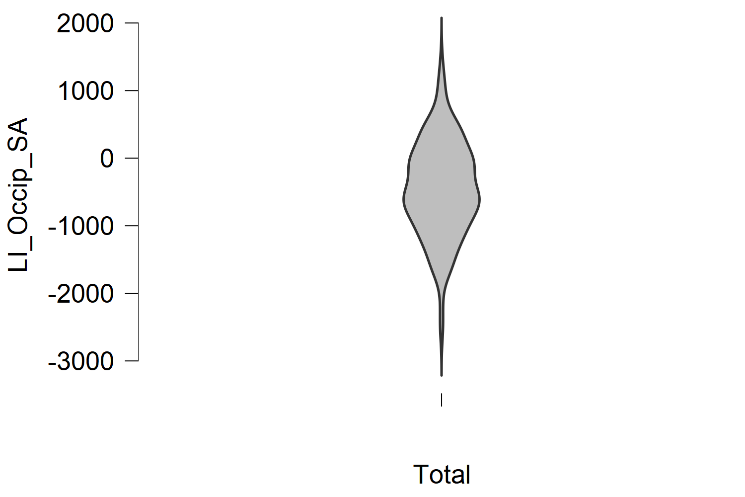


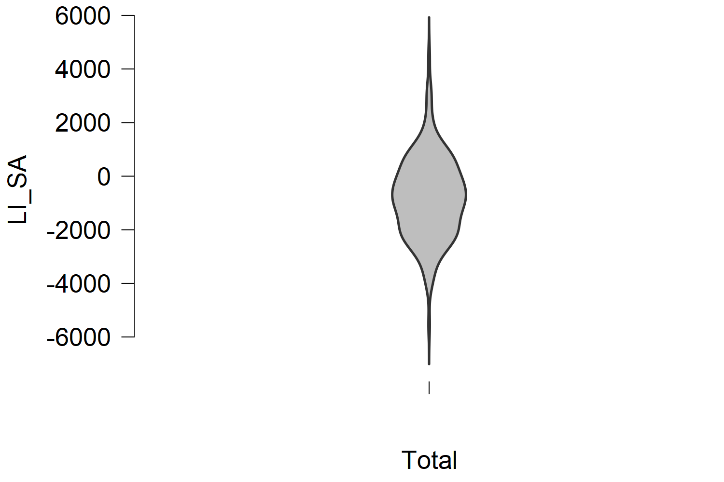


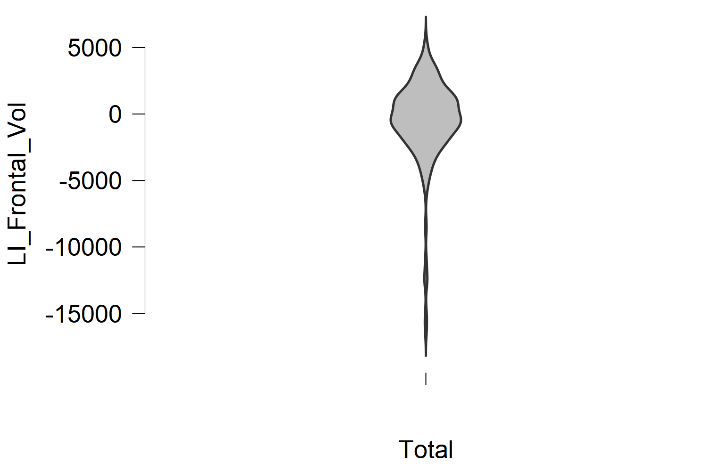

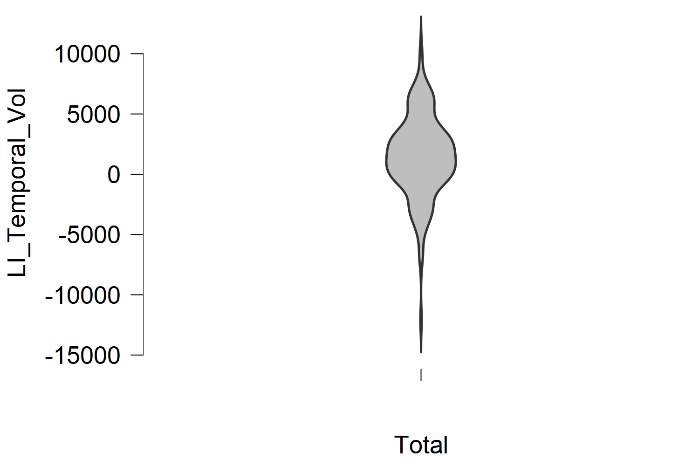


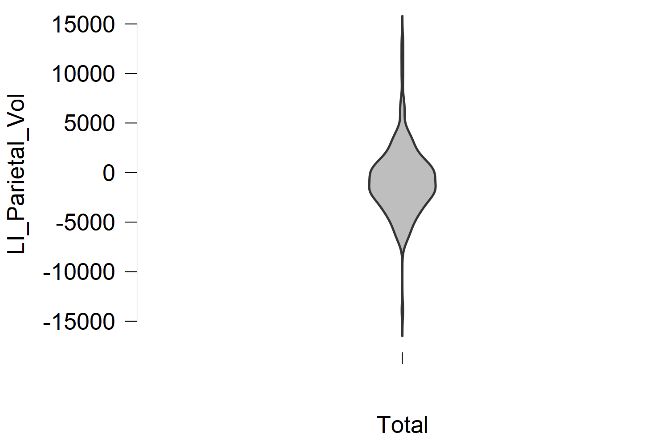

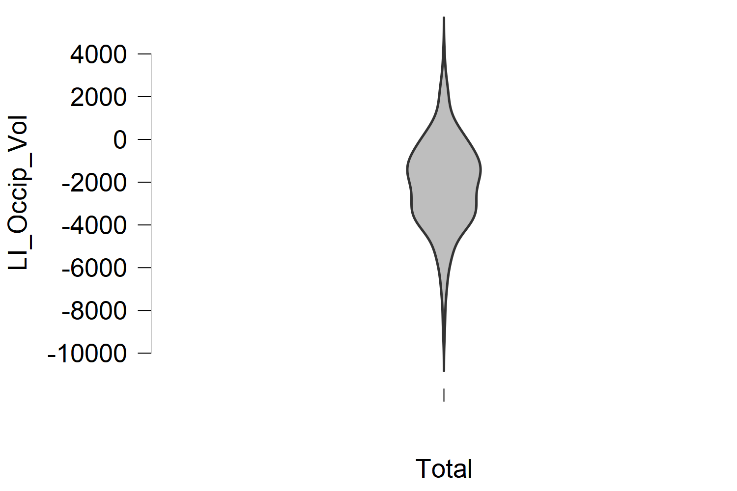


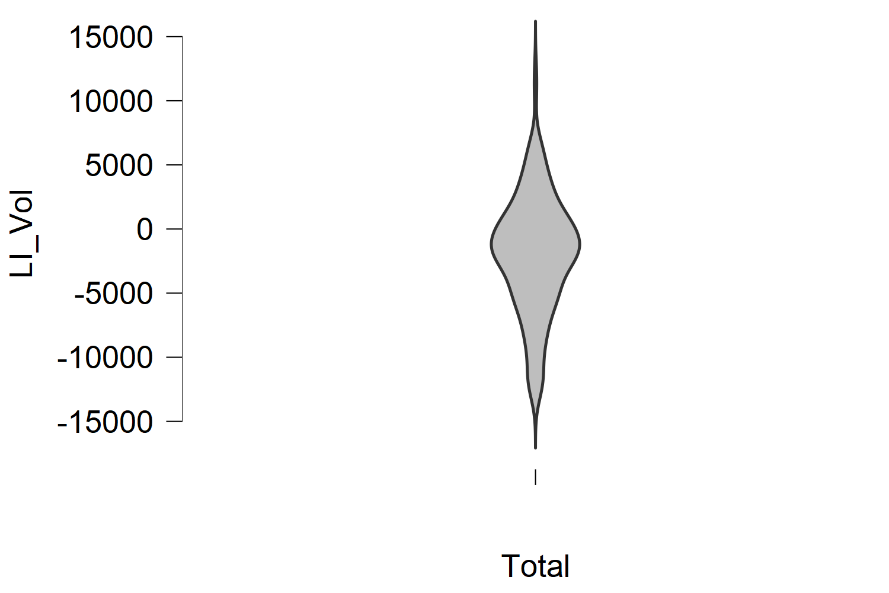


LI= Lateralization Index, the difference between left hemisphere/lobe and right hemisphere/lobe. SA= Surface Area, Vol= Volume

| **Supplementary table 5**. Regression models for cortical surface areas of the regions of interest | | | | |  |  |
| --- | --- | --- | --- | --- | --- | --- |
|  |  |  |  |  |  |  |
| Region of interest |  | Unstardardized Coefficients | | Standardized Coefficients |  |  |
|  |  | B | Std.error | Beta | t | p |
| Left hemisphere | ΔR2 = 0.233 |  |  |  |  |  |
|  | Intercept | 92548.854 |  |  | 108.068 | <0.001 |
|  | Sex | -6394.823 | 1231.148 | -0.454 | -5.194 | <0.001 |
|  | NICU stay | 4296.865 | 2159.834 | 0.174 | 1.989 | 0.049 |
| Right hemisphere | ΔR2 = 0.270 |  |  |  |  |  |
|  | Intercept | 93898.948 | 878.226 |  | 106.919 | <0.001 |
|  | Sex | -6870.241 | 1239.729 | -0.473 | -5.542 | <0.001 |
|  | NICU stay | 5220.45 | 2203.441 | 0.205 | 2.369 | 0.020 |
|  | Smoking | -4827.973 | 2320.645 | -0.179 | -2.080 | 0.040 |
| Left frontal | ΔR2 = 0.198 |  |  |  |  |  |
|  | Intercept | 34632.929 | 341.152 |  | 101.518 | <0.001 |
|  | Sex | -2590.472 | 508.006 | -0.454 | -5.099 | <0.001 |
| Right frontal | ΔR2 = 0.167 |  |  |  |  |  |
|  | Intercept | 35182.821 | 389.038 |  | 90.436 | <0.001 |
|  | Sex | -2669.430 | 579.312 | -0.418 | -4.608 | <0.001 |
| Left temporal | ΔR2 = 0.136 |  |  |  |  |  |
|  | Intercept | 17864.661 | 171.617 |  | 104.096 | <0.001 |
|  | Sex | -1049.182 | 255.554 | -0.380 | -4.106 | <0.001 |
| Right temporal | ΔR2 = 0.212 |  |  |  |  |  |
|  | Intercept | 17647.327 | 172.592 |  | 102.249 | <0.01 |
|  | Sex | -1270.74 | 249.794 | -0.450 | -5.087 | <0.001 |
|  | Smoking | -949.658 | 462.324 | -0.182 | -2.054 | 0.043 |
| Left parietal | ΔR2 = 0.224 |  |  |  |  |  |
|  | Intercept | 28221.694 | 307.444 |  | 91.795 | <0.001 |
|  | Sex | -2011.924 | 433.996 | -0.408 | -4.636 | <0.001 |
|  | NICU stay | 2006.496 | 771.366 | 0.232 | 2.601 | 0.011 |
|  | Smoking | -1700.972 | 812.36 | -0.186 | -2.094 | 0.039 |
| Right parietal | ΔR2 = 0.286 |  |  |  |  |  |
|  | Intercept | 33269.109 | 2957.655 |  | 11.248 | <0.001 |
|  | Sex | -2091.949 | 393.354 | -0.448 | -5.318 | <0.001 |
|  | Maternal BMI before gestation | 82.173 | 44.989 | 0.157 | 1.827 | 0.071 |
|  | Smoking | -2137.658 | 745.459 | -0.247 | -2.867 | 0.005 |
|  | 5min APGAR score | -718.263 | 274.478 | -0.229 | -2.617 | 0.010 |
| Left occipital | ΔR2 = 0.098 |  |  |  |  |  |
|  | Intercept | 12226.839 | 170.999 |  | 71.502 | 0.010 |
|  | Sex | -880.209 | 254.633 | -0.327 | -3.457 | 0.010 |
| Right occipital | ΔR2 = 0.113 |  |  |  |  |  |
|  | Intercept | 12785.036 | 191.373 |  | 66.807 | 0.010 |
|  | Sex | -1060.541 | 284.972 | -0.349 | -3.721 | 0.010 |
|  |  |  |  |  |  |  |
| BMI= body mass index, NICU= neonatal intensive care unit | | |  |  |  |  |

| **Supplementary table 6.** Regression models for cortical volumes of the regions of interest | | | |  |  |  |
| --- | --- | --- | --- | --- | --- | --- |
|  |  |  |  |  |  |  |
| Region of interest | Predictor | Unstardardized Coefficients | | Standardized Coefficients | . |  |
|  |  | B | Std.error | Beta | t | p |
| Left hemisphere | ΔR2 = 0.180 |  |  |  |  |  |
|  | Intercept | 295874.645 | 6411.987 |  | 46.144 | <0.001 |
|  | Sex | -15443.847 | 3803.134 | -0.367 | -4.061 | <0.001 |
|  | Maternal education | 9291.872 | 3787.726 | 0.222 | 2.453 | 0.016 |
| Right hemisphere | ΔR2 = 0.176 |  |  |  |  |  |
|  | Intercept | 281648.405 | 13146.871 |  | 21.423 | <0.001 |
|  | Sex | -16929.853 | 3973.653 | -0.385 | -4.261 | <0.001 |
|  | Maternal age at child's birth | 1020.654 | 424.560 | 0.217 | 2.404 | 0.018 |
| Left frontal | ΔR2 = 0.211 |  |  |  |  |  |
|  | Intercept | 116746.722 | 2566.493 |  | 45.489 | <0.001 |
|  | Sex | -7205.936 | 1522.260 | -0.419 | -4.734 | <0.001 |
|  | Maternal education | 3367.145 | 1516.093 | 0.197 | 2.221 | 0.029 |
| Right frontal | ΔR2 = 0.148 |  |  |  |  |  |
|  | Intercept | 122564.875 | 1151.887 |  | 106.404 | <0.001 |
|  | Sex | -7394.897 | 1715.264 | -0.396 | -4.311 | <0.001 |
| Left temporal | ΔR2 = 0.076 |  |  |  |  |  |
|  | Intercept | 64528.682 | 1683.306 |  | 38.334 | <0.001 |
|  | Maternal education | 2348.389 | 994.372 | 0.226 | 2.362 | 0.020 |
|  | Sex | -2001.561 | 998.417 | -0.192 | -2.005 | 0.048 |
| Right temporal | ΔR2 = 0.110 |  |  |  |  |  |
|  | Intercept | 63038.236 | 1810.103 |  | 34.826 | <0.001 |
|  | Sex | -2871.587 | 1073.624 | -0.252 | -2.675 | 0.009 |
|  | Maternal education | 2682.296 | 1069.274 | 0.236 | 2.509 | 0.014 |
| Left parietal | ΔR2 = 0.126 |  |  |  |  |  |
|  | Intercept | 83938.651 | 2377.323 |  | 35.308 | <0.001 |
|  | Sex | -4449.082 | 1410.059 | -0.294 | -3.155 | 0.002 |
|  | Maternal education | 3273.144 | 1404.346 | 0.217 | 2.331 | 0.002 |
| Right parietal | ΔR2 = 0.205 |  |  |  |  |  |
|  | Intercept | 73520.942 | 4310.590 |  | 17.056 | <0.001 |
|  | Sex | -4310.438 | 1248.428 | -0.307 | -3.453 | <0.001 |
|  | Maternal age at child's birth | 375.256 | 134.865 | 0.250 | 2.782 | 0.006 |
|  | Maternal education | 3009.091 | 1260.523 | 0.215 | 2.387 | 0.019 |
| Left occipital | ΔR2 = 0.057 |  |  |  |  |  |
|  | Intercept | 31131.625 | 455.341 |  | 68.370 | <0.001 |
|  | Sex | -1810.103 | 678.044 | -0.258 | -2.670 | 0.009 |
| Right occipital | ΔR2 = 0.043 |  |  |  |  |  |
|  | Intercept | 33251.786 | 517.972 |  | 64.196 | <0.001 |
|  | Sex | -1823.090 | 771.307 | -0.230 | -2.364 | 0.020 |
